# Impaired resolution of blood transcriptomes through tuberculosis treatment with diabetes comorbidity

**DOI:** 10.1002/ctm2.1375

**Published:** 2023-08-30

**Authors:** Clare Eckold, Cassandra L. R. van Doorn, Rovina Ruslami, Katharina Ronacher, Anca‐Lelia Riza, Suzanne van Veen, Ji‐Sook Lee, Vinod Kumar, Sarah Kerry‐Barnard, Stephanus T. Malherbe, Léanie Kleynhans, Kim Stanley, Simone A. Joosten, Julia A Critchley, Philip C. Hill, Reinout van Crevel, Cisca Wijmenga, Mariëlle C. Haks, Mihai Ioana, Bachti Alisjahbana, Gerhard Walzl, Tom H. M. Ottenhoff, Hazel M. Dockrell, Eleonora Vianello, Jacqueline M. Cliff, the TANDEM Consortium$

**Affiliations:** ^1^ Department of Infection Biology and TB Centre London School of Hygiene & Tropical Medicine London UK; ^2^ Department of Infectious Diseases Leiden University Medical Center Leiden The Netherlands; ^3^ Department of Biomedical Sciences Faculty of Medicine Universitas Padjadjaran Bandung Indonesia; ^4^ DSI‐NRF Centre of Excellence for Biomedical Tuberculosis Research South African Medical Research Council Centre for Tuberculosis Research Division of Molecular Biology and Human Genetics Department of Biomedical Sciences Faculty of Medicine and Health Sciences Stellenbosch University Cape Town South Africa; ^5^ Mater Research Institute Faculty of Medicine Translational Research Institute The University of Queensland Brisbane QLD Australia; ^6^ Department of Internal Medicine and Radboud Center for Infectious Diseases Radboud University Medical Center Nijmegen The Netherlands; ^7^ Human Genomics Laboratory Department of Diagnostics and Treatment University of Medicine and Pharmacy of Craiova Craiova Romania; ^8^ Regional Centre for Human Genetics – Dolj Emergency Clinical County Hospital Craiova Craiova Romania; ^9^ Department of Genetics University of Groningen University Medical Center Groningen Groningen The Netherlands; ^10^ Population Health Research Institute St George's University of London London UK; ^11^ Division of Health Sciences Centre for International Health University of Otago Dunedin New Zealand; ^12^ Nuffield Department of Medicine Centre for Tropical Medicine and Global Health University of Oxford Oxford UK; ^13^ Internal Medicine Department Hasan Sadikin General Hospital Bandung Indonesia; ^14^ Research Center for Care and Control of Infectious Diseases Universitas Padjadjaran Bandung Indonesia; ^15^ Department of Life Sciences Centre for Inflammation Research and Translational Medicine Brunel University London London UK

**Keywords:** diabetes, transcriptome, treatment, tuberculosis

## Abstract

**Background:**

People with diabetes are more likely to develop tuberculosis (TB) and to have poor TB‐treatment outcomes than those without. We previously showed that blood transcriptomes in people with TB‐diabetes (TB‐DM) co‐morbidity have excessive inflammatory and reduced interferon responses at diagnosis. It is unknown whether this persists through treatment and contributes to the adverse outcomes.

**Methods:**

Pulmonary TB patients recruited in South Africa, Indonesia and Romania were classified as having TB‐DM, TB with prediabetes, TB‐related hyperglycaemia or TB‐only, based on glycated haemoglobin concentration at TB diagnosis and after 6 months of TB treatment. Gene expression in blood at diagnosis and intervals throughout treatment was measured by unbiased RNA‐Seq and targeted Multiplex Ligation‐dependent Probe Amplification. Transcriptomic data were analysed by longitudinal mixed‐model regression to identify whether genes were differentially expressed between clinical groups through time. Predictive models of TB‐treatment response across groups were developed and cross‐tested.

**Results:**

Gene expression differed between TB and TB‐DM patients at diagnosis and was modulated by TB treatment in all clinical groups but to different extents, such that differences remained in TB‐DM relative to TB‐only throughout. Expression of some genes increased through TB treatment, whereas others decreased: some were persistently more highly expressed in TB‐DM and others in TB‐only patients. Genes involved in innate immune responses, anti‐microbial immunity and inflammation were significantly upregulated in people with TB‐DM throughout treatment. The overall pattern of change was similar across clinical groups irrespective of diabetes status, permitting models predictive of TB treatment to be developed.

**Conclusions:**

Exacerbated transcriptome changes in TB‐DM take longer to resolve during TB treatment, meaning they remain different from those in uncomplicated TB after treatment completion. This may indicate a prolonged inflammatory response in TB‐DM, requiring prolonged treatment or host‐directed therapy for complete cure. Development of transcriptome‐based biomarker signatures of TB‐treatment response should include people with diabetes for use across populations.

## Graphical abstract

Aberrant blood transcriptomes in people with tuberculosis are further altered in people who also have type 2 diabetes.

Excessive inflammatory responses persist throughout tuberculosis treatment in tuberculosis‐diabetes co‐morbid patients.

Inclusion of TB patients with diabetes or elevated hyperglycaemia permits the development of biosignatures which accurately predict tuberculosis‐treatment response across the normoglycaemia/diabetes spectrum and across geographical populations.

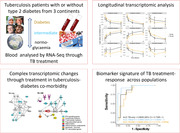

## 1 INTRODUCTION

Diabetes mellitus (DM) negatively impacts on tuberculosis (TB) control efforts by increasing the risk of *Mycobacterium tuberculosis* infection^1^ and of progression to active TB disease three‐fold.^2^, ^3^ The growing prevalence of DM, particularly in countries with high burdens of TB, means DM now underlies around 15% of TB cases globally,^4^ accounting for 10% of TB deaths in HIV‐negative people. Concomitant DM negatively affects TB‐treatment outcomes and is associated with increased risks of delayed sputum conversion, relapse, treatment failure and death: the relative risk for each poor outcome is ∼2 to ∼5 in meta‐analyses.^5^, ^6^ It is unknown whether extending standard TB treatment would improve the outcome for TB‐DM comorbid patients, or whether alternative treatment is required, such as host‐directed therapy. Improvement of diabetes management in people with TB‐ DM comorbidity may also improve TB outcomes. A pragmatic clinical study^7^ linked to this one showed that structured DM monitoring and intervention improved glycaemic control in TB‐DM patients, but was underpowered to determine the effect on TB treatment outcome.

The worldwide DM prevalence is ∼463 million people and is estimated to rise to 700 million by 2045.^8^ The majority of people have type‐2 DM, caused by a reduction in the response to insulin thereby reducing its ability to control target cell metabolism, which triggers an increase in insulin production leading to pancreatic damage through exhaustion, and impaired glucose tolerance. There is a spectrum from normal through to overt DM via intermediate hyperglycaemia (IH), and people with IH are more likely to develop DM in the future.^9^ As well as measures such as impaired fasting glucose and the impaired glucose tolerance test, the HbA1c concentration can indicate an individual's position on this spectrum.^9^ Infectious diseases, including TB, can cause temporary stress hyperglycaemia, which carries a higher risk of adverse events than longer‐term pre‐diabetes.^10^ TB‐induced stress hyperglycaemia also makes DM diagnosis difficult: some people with apparent newly diagnosed DM at TB diagnosis no longer reach DM diagnostic criteria after TB treatment.^11^ TB incidence and TB‐DM treatment outcomes are worse in people with poorly controlled DM with higher HbA1c concentrations.^12^

People with TB‐DM comorbidity have altered immunity compared with people with uncomplicated TB, with both innate and adaptive immune responses affected.^13^ In plasma, various inflammatory cytokines such as IL‐1β, IL‐17A, interferon (IFN)γ and TNFα are more elevated in people with TB‐DM^14^, ^15^ and TB‐pre‐diabetes^16^ than in people with uncomplicated TB. People with TB‐DM have more circulating Th1 and Th17 cells and fewer Tregs. In uncomplicated TB, peripheral immune responses typically resolve to normal levels during successful TB treatment.^15^ In contrast, the excessive inflammatory plasma cytokine responses in TB‐DM are still evident after treatment completion,^17^ and dendritic cell, monocyte^18^ and T cell differentiation^19^ aberrations are still present at 2 months, although resolved by 6 months, indicating a delayed response to TB treatment in TB‐DM patients.

Transcriptomic technologies have shown widespread changes in gene expression occur in blood from TB patients compared with healthy individuals in multiple studies, revealing an enhanced circulating inflammatory and type 1 IFN response.^20^, ^21^, ^22^, ^23^ With successful TB treatment, this transcriptomic signature is rapidly downregulated, has largely diminished after 2 months of treatment and mostly disappears by 12 months,^21^, ^23^, ^24^ mirroring clinical resolution and chest X‐ray improvement; however, transcriptomes do not fully resolve with poor TB treatment outcome,^25^ including in people with TB‐DM comorbidity.^26^ We recently showed^27^ that DM comorbidity, as well as IH, significantly affects the TB diagnosis biosignature, causing an enhanced inflammatory but reduced type 1 IFN response. This is in concordance with reduced IFNβ responses to Toll‐like receptor stimulation in people with DM.^28^ Differences in the changes in blood transcriptomes through TB treatment between people with TB‐DM comorbidity and those with uncomplicated TB have not been described. The main aim of this study was to determine whether transcriptomic biosignatures resolve normally in people with TB‐DM co‐morbidity, or whether changes during TB treatment are kinetically or qualitatively different to those observed in people with uncomplicated TB alone. Additionally, differences in TB patients with pre‐diabetes or IH compared with uncomplicated were identified. The characterisation of any such differences between people with TB‐DM and TB‐only may indicate the underlying mechanisms for worse TB treatment outcomes and may indicate promising avenues for the development of new therapies.

## 2 METHODS

### 2.1 Patient recruitment and classification

Newly diagnosed patients with bacteriologically confirmed pulmonary TB, with or without concomitant DM, were recruited in three locations: Bandung, Indonesia (UNPAD), Cape Town, South Africa (SUN) and Craiova, Romania (UMFCV), as part of the TANDEM project.^29^ Exclusion criteria were multi‐drug‐resistant TB, HIV positivity, pregnancy, other serious co‐morbidity or corticosteroid use. In South Africa, healthy controls (HCs) without TB, diabetes or hyperglycaemia were also enrolled: all had laboratory HbA1c < 5.7% were sputum smear and culture negative and had normal chest X‐rays. Samples were not available from HCs in the Indonesian cohort. All participants gave written informed consent. The study was approved by the LSHTM Observational Research Ethics Committee (6449/July2013), the UNPAD Health Research Ethics Committee (377/UN6.C2.1.2/ KEPK/PN/2012), the SUN Health Research Ethics Committee (N13/05/064/July2013) and the UMFCV Committee of Ethics and Academic and Scientific Deontology (94/September2013).

**FIGURE 1:**
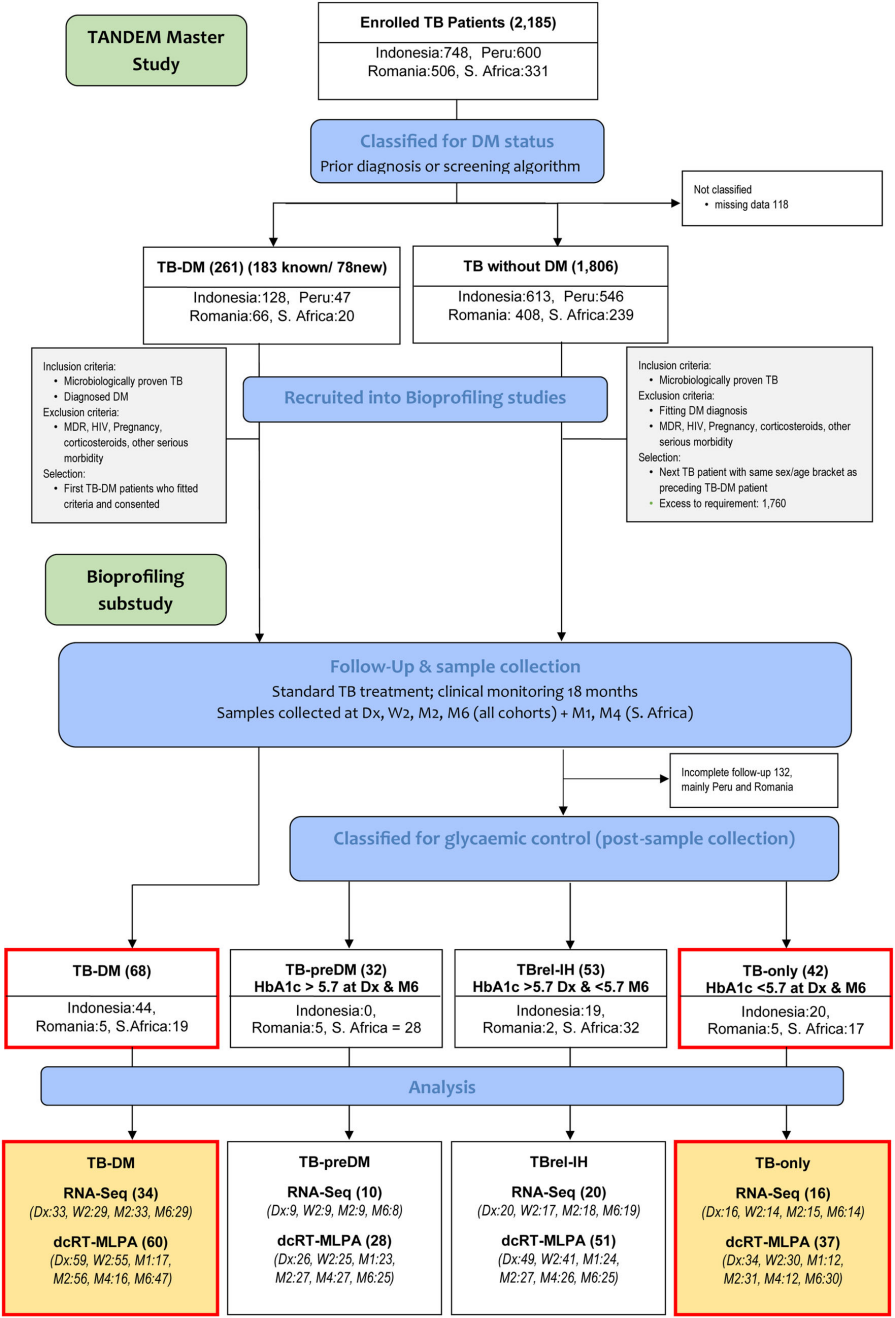
Recruitment of participants with TB into the TANDEM study, and selection of participants for inclusion in the gene expression analyses. The TANDEM study was a multi‐centre, multidisciplinary project investigating various factors in TB and diabetes co‐morbidity. This bioprofiling study was nested within the TANDEM Master study, in which 2185 TB patients were recruited to undergo screening for diabetes. They were initially classified into those with diabetes or without diabetes and were recruited into the bioprofiling sub‐study if they met the inclusion and exclusion criteria. Study participants were followed up at time points shown, with blood samples taken for gene expression analyses. The primary aim was to compare people with TB and with TB‐DM comorbidity through TB treatment. Secondarily, we analysed gene expression in TB patients with stable (TB‐preDM) or transient (TBrel‐IH) elevated glycaemia, as we discovered that this also impacted gene expression.

All TB patients underwent first line TB treatment according to WHO guidelines. Most patients diagnosed with DM received the local standard of care treatment, and the medication taken was noted. A TB‐DM subgroup within the Indonesian cohort had intensive HbA1c monitoring as part of a pragmatic randomised control trial, with DM medication changed accordingly.^7^ Participants were classified by DM/glycaemia status at TB diagnosis and after 6 months of TB treatment (Figure 1 and Table S1). The ‛TB‐DM’ group included patients with both pre‐existing and newly diagnosed DM (Table S2). People with newly diagnosed TB‐DM had laboratory HbA1c test ≥6.5% with confirmatory HbA1c test ≥6.5% or fasting blood glucose ≥7 mmol/L at TB diagnosis,^29^, ^30^ followed by a further HbA1c test ≥6.5% after 6 months of TB treatment. Infection, including TB, can drive impairments in glucose control leading to elevated HbA1c, which can then resolve when the infection is cleared. In order to distinguish between people with TB‐induced, transiently elevated HbA1c from people who had pre‐diabetes irrespective of their TB, we further sub‐classified people who had HbA1c ≥5.7% at TB diagnosis. TB patients whose HbA1c test results were ≥5.7% and <6.5% at both TB diagnosis and at 6 months were deemed to have pre‐diabetes (‛TB‐preDM’). Patients whose HbA1c result was ≥5.7% at TB diagnosis but <5.7% at 6 months were deemed to have TB‐related IH at TB diagnosis (‛TBrel‐IH’).

### 2.2 Sample collection and RNA extraction

Venous blood samples (2.5 mL) were collected into PAXgene Blood RNA Tubes (PreAnalytiX) from TB patients prior to TB treatment initiation (W0) and at intervals through treatment (W2,4,8,16,26) up to 12 months post diagnosis (W52) and stored at −80°C prior to analysis. Total RNA was extracted using RNeasy spin columns (Qiagen) and quantified by Nanodrop (Agilent).

### 2.3 Unbiased whole genome RNA‐Seq

RNA samples that were processed for RNA‐Seq analysis were quality‐assessed using the LabChip GX HiSens RNA system (PerkinElmer). Total RNA samples were processed using the poly‐A tail Bioscientific NEXTflex‐Rapid‐Directional mRNA‐seq method with the Caliper SciClone to generate libraries, which were single‐end sequenced using the NextSeq500 High Output kit V2 (Illumina) for 75 cycles. Data are deposited in the NCBI‐GEO database, accession number GSE193978. STAR (v2.5.1b)^31^ was used to align the sequence data from FASTQ files to the Human g1kv37 reference genome, and quality control was performed with FastQC.^32^ Downstream data analysis was performed in R.^33^ HTseq‐count (v0.61) was used for transcript quantification,^34^ and lowly abundant transcripts were removed. Data were normalised using the DESeq2 (1.30.0)^35^ R package, which included a correction for sex.

For the MaSigPro^36^ analysis, due to the number of timepoints, a quadratic regression model (degrees of freedom = 2) was executed. MaSigPro uses a two‐step regression‐based approach which finds genes with temporal differences and also differences between groups. It is similar to a two‐way ANOVA but for longitudinal RNA‐Seq data, accounting for similarity between samples from the same individual. This includes an initial least‐squares technique and then stepwise regression. The coefficients obtained then undergo hierarchical clustering to group the genes together that behave similarly. False discovery correction was done using the Benjamini–Hochberg method with an adjusted *p* value < .05 deemed to be significant. From the genes that were found to be differentially expressed between clinical groups, the R package tmod^37^ and its HGtest function were used for modular analysis, with all genes used as the background. Modules with an adjusted *p* value < .05 were deemed significant. Modular activity was calculated by summing the differential expression of genes in the TB‐DM group relative to the TB‐only group within a module and then dividing by the number of genes within that module. Molecular degree of perturbation (MDP) analysis was performed using the R package mdp.^38^ The g:profiler webtool^39^ was used for gene ontology and pathway analyses of gene lists.

### 2.4 Targeted gene expression profiling

Reverse‐Transcriptase Multiplex Ligation‐dependent Probe Amplification (dcRT‐MLPA) was performed using the SALSA MLPA kit (MRC‐Holland) as described elsewhere.^40^ RT primers and half‐probes were designed by Leiden University Medical Centre (LUMC, Leiden, the Netherlands)^41^, ^42^ and included sequences for four housekeeping genes and 144 selected key immune‐related genes to profile specific compartments of the human immune response (Table S3): (1) innate immune response: inflammasome components, pattern recognition receptors and myeloid‐associated genes; (2) inflammatory and IFN‐signalling genes (ISGs); (3) adaptive immune response: T, Th, Treg, B and NK cell markers; (4) other genes: anti‐microbial activity, inflammation, intracellular transport, markers of apoptosis, cell survival or cell growth, proliferation and activation, additional chemokines, transcriptional regulators/activators. Briefly, 125 ng RNA was reverse transcribed to cDNA by incubation at 37°C for 15 min using Moloney Murine Leukemia Virus reverse transcriptase (Promega) with gene‐specific RT‐primers (Sigma–Aldrich), followed by inactivation of the enzyme by heating at 98°C for 2 min. The left‐ and right‐hand half probes were hybridised to the cDNA at 60°C overnight, followed by ligation at 54°C for 15 min using ligase‐65 (MRC‐Holland), and inactivation by heating at 98°C for 5 min. Ligated probes were amplified by PCR (33 cycles at 95°C for 30 s, 58°C for 30 s and 72°C for 60 s, followed by one cycle at 72°C for 20 min). PCR products were 1:10 diluted in Highly deionised (Hi‐Di) formamide (ThermoFisher) containing the 400HD Rhodamine X (ROX) fluorophore size standard (ThermoFisher). PCR products were denatured at 95°C for 5 min, stored immediately at 4°C and analysed on an Applied Biosystems 3730 capillary sequencer in GeneScan mode (BaseClear). Trace data were analysed using GeneMapper software 5 (Applied Biosystems). The areas of each assigned peak (arbitrary units) were exported for analysis in R (version 3.6.3). Data were normalised to the housekeeping gene glyceraldehyde 3‐phosphate dehydrogenase (GAPDH) and signals below the threshold value for noise cutoff in GeneMapper (log2 transformed peak area 7.64) were assigned the threshold value for noise cutoff.

dcRT‐MLPA data were analysed to identify differentially expressed genes (DEGs) between groups at diagnosis by the non‐parametric Mann–Whitney *U*‐test with Benjamini–Hochberg correction for multiple testing. Ingenuity pathway analysis (IPA‐60467501) (QIAGEN) was used to explore interactive networks between the DEGs. MDP analysis (mdp R package),^38^ partial least squares—discriminant analysis (PLS‐DA) (mixOmics R package)^43^ and Pearson correlations of gene expression (log2 FC) versus HC were performed in R version 4.0.2. Longitudinal changes in gene expression levels from diagnosis (baseline) to 6 months (Indonesian cohort) or 12 months (South African cohort) were assessed by means of linear mixed models for repeated measurements over time. Models were fitted to Log_2_‐transformed measurements in the lme4 R package using the lmer function.^44^ Group–time interactions were included as fixed effects and the patient identifier–time interactions were included as random effects. For the South African cohort, we forced a b‐spline at 6 months, which enabled us to identify altered gene expressions during treatment (0–6 months) as well as altered gene expression after treatment (6–12 months). Time was coded as 0 for the first timepoint (diagnosis) and as a continuous variable for the time difference between the two time points. *p* Values were adjusted for multiple testing using the false discovery rate method of Benjamini–Hochberg.^45^ An adjusted *p* value < .05 and a log2‐fold change (FC) <− .6 and > .6 were set as thresholds for the identification of DEGs. Genes that were below the detection limit in >90% of the samples per cohort were excluded from the analysis. Signatures with the best discriminatory capability were identified using logistic regression with lasso regularisation (glmnet R package).^46^ Leave‐one‐out cross validation and train‐test split were used to assess the performance of the trained regression models. The classifying performance of the models were assessed by evaluating the sensitivity, specificity, receiver operating characteristic (ROC) curve and area under the ROC curve (AUC) with 95% confidence interval (CI), and box‐and‐whiskers‐plots representing the predicted probability for each class were used to evaluate the classifying performance of the models. Ingenuity pathway analysis was used to identify top canonical pathways, upstream regulators and causal networks in the biosignature model and MaSigPro RNASeq DEG lists. Therapeutic drug inhibitors were identified using the Therapeutic Target Database^47^ and the GeneCards Database.^48^

## 3 RESULTS

### 3.1 Study population

TB patients were recruited in South Africa, Indonesia and Romania, as a nested sub‐study within the TANDEM project^29^ (Figure 1 and Table S1). Sixty‐eight patients had TB and DM comorbidity, of whom 49 had pre‐existing diabetes and 19 were diagnosed upon study recruitment (Table S2). Forty‐two TB patients without diagnosed DM were classified as having uncomplicated TB‐only, whereas 53 had TBrel‐IH at the time of TB diagnosis which resolved by the end of treatment. Thirty‐two patients had TB‐preDM, with persistent moderately elevated glycaemia. TB patients were all followed up for 18 months, with blood samples collected during TB treatment. In South Africa, HCs (*n* = 27) were also recruited, with blood samples collected at one time. Their RNASeq profiles have been published previously,^27^ and they are included here in the targeted gene dcMLPA analysis for reference. All groups were evenly sex balanced, except for male predominance in the TB‐PreDM group. Age ranges were similar across clinical groups. In the Indonesian TB‐DM group, there was a highly significant decrease in HbA1c through TB treatment, which was likely due to the intensive DM follow‐up in Bandung (Figure S1 and Table S1); this was not evident in South Africa or Romania.

### 3.2 Global longitudinal transcriptomes in TB‐DM

**FIGURE 2:**
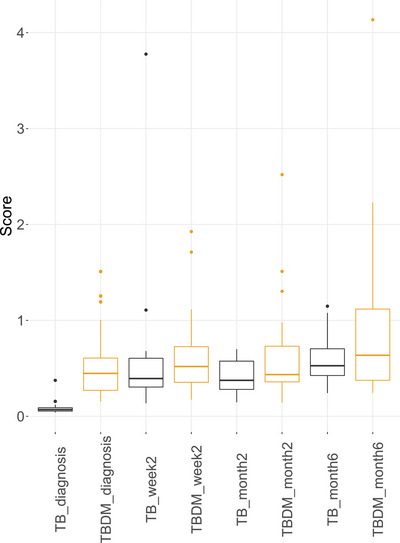
Molecular degree of perturbation plots representing change in global gene expression in blood relative to patients with TB‐only at TB diagnosis. Gene expression was determined by RNA‐Seq of whole venous blood from pulmonary TB patients from all three clinical locations with (TB‐DM: *n* = 34) or without (TB‐only: *n* = 18) concomitant diabetes, at TB diagnosis and during TB treatment. The bars show the median and 1.5*inter‐quartile range.

Gene expression was determined in venous blood by RNA‐Seq in a subgroup of study participants from the four TB patient clinical groups (TB‐DM: *n* = 34; TB‐PreDM: *n* = 10; TBrel‐IH: *n* = 20; TB‐only: *n* = 16; Table S1 and Figure 1). The MDP of gene expression in individual samples from patients with TB‐only or TB‐DM over time was calculated relative to the mean gene expression at diagnosis in people with TB‐only (Figure 2). We have previously shown that the gene expression in TB and TB‐DM patients at TB diagnosis is perturbed relative to HCs.^27^ The overall gene expression was different between TB and TB‐DM patients at diagnosis. As expected, there were changes in gene expression during TB treatment in the TB‐only group, which were evident by week 2 and continued throughout treatment. The global gene expression change in the TB‐DM group through treatment was of lower magnitude, indicating less impact of TB treatment: global gene expression in the TB‐DM group remained different to the TB‐only group at all time points (Figure 2).

**FIGURE 3:**
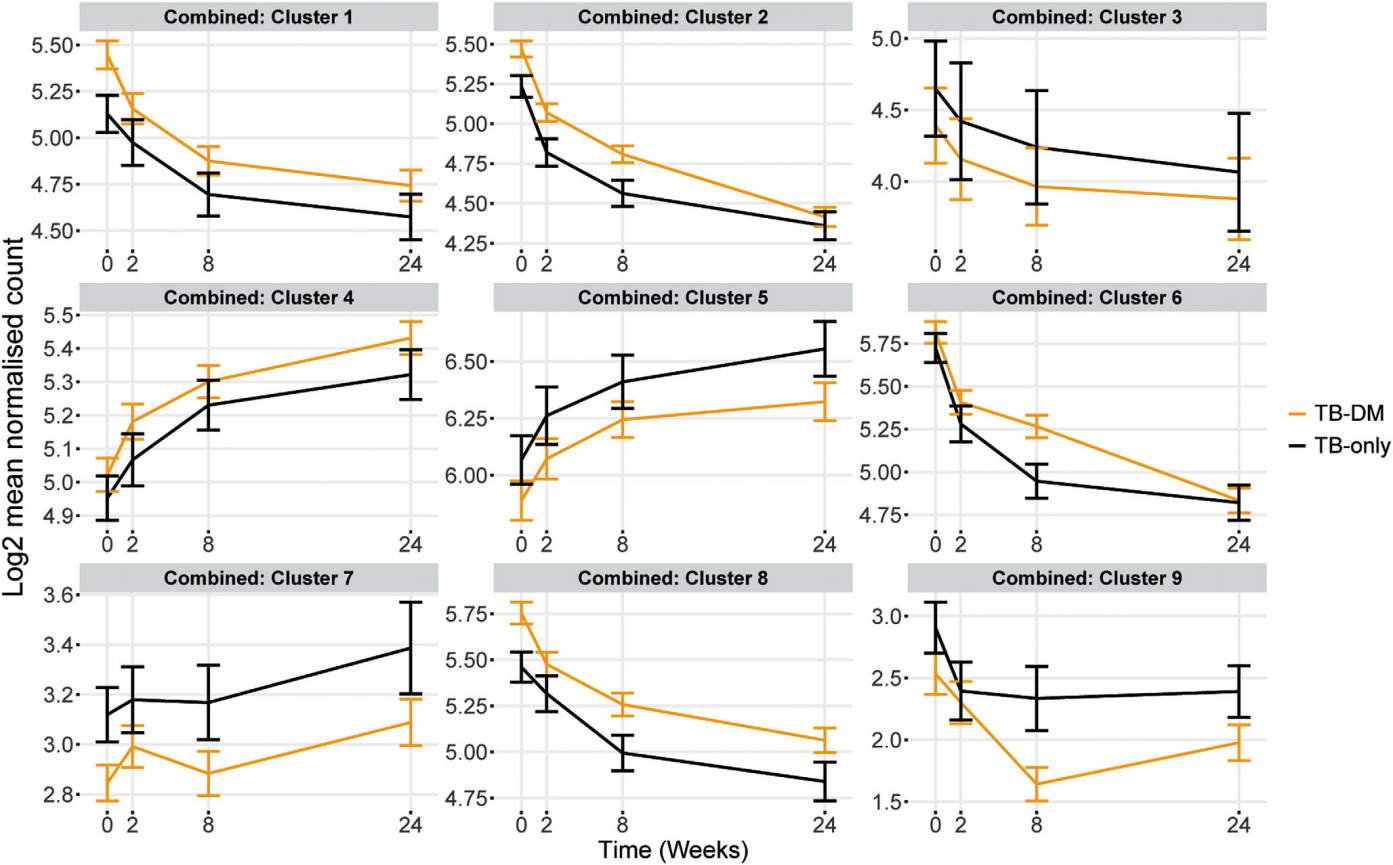
MaSigPro analysis of change in gene expression through TB treatment in blood samples from patients in all three populations combined (South Africa, Indonesia and Romania). MaSigPro identified genes that behave similarly between patient groups using hierarchical clustering. Results are shown for log‐transformed normalised count for the TB‐only group or TB‐DM. Bars show mean ± 1 SEM. Data were filtered to remove lowly abundant transcripts prior to analysis.

The MaSigPro package performs a two‐step regression analysis. This novel statistical approach identifies genes that change significantly across groups and through time. Traditional methods rely on pairwise comparisons which would be ineffective at capturing the dynamic nature of longitudinal data.^36^ This analysis identified 167 genes with significantly different changed expression between TB‐DM and TB‐only groups through TB treatment, in the combined dataset from South Africa, Indonesia and Romania. Hierarchical clustering of these genes based on similar expression patterns yielded nine clusters (Figure 3 and Table S4). Clusters which were more highly expressed in TB‐DM patients throughout treatment (clusters 1, 2, 4 and 8) were enriched for genes involved in the innate immune response, IL‐4 signalling, protein dimerisation and neutrophil chemotaxis, determined using the DAVID Functional Annotation Tool^49^ (Table 1). Cluster 6 exhibited divergence between TB and TB‐DM patients only at week 8 of treatment: this cluster was enriched for genes involved in anti‐viral and IFN signalling responses. Clusters more highly expressed in TB‐only patients (clusters 3, 5, 7 and 9) were smaller and enriched for alternative splice variants and immunoglobulins.

**TABLE 1:** Clusters of genes differentially expressed between TB‐DM and TB‐only patients in MaSigPro analysis of the combined RNA‐Seq dataset from South Africa, Indonesia and Romania.

			Gene function				
Cluster	Overall pattern	Number of transcripts	Protein coding	Processed transcript	Pseudo‐gene	Regulatory RNAs^b^	Top non‐redundant functions from DAVID^a^
1	Higher in TB‐DM throughout; decreasing with time	27	19	0	2	6	Innate Immunity Antimicrobial RAGE receptor binding
2	Higher in TB‐DM throughout; decreasing with time	50	28	5	4	13	IPAF inflammasome IL‐4 signalling Transmembrane helices
3	Lower in TB‐DM throughout; decreasing with time	6	6	0	0	0	Disulphide bond Inflammation/fibrosis
4	Higher in TB‐DM throughout; increasing with time	17	11	3	0	3	Coiled coil Protein homo‐dimersiation
5	Lower in TB‐DM throughout; increasing with time	9	8	1	0	0	Collagen‐binding Alternative splicing phosphoprotein
6	Higher in TB‐DM at week 8, otherwise similar	28	22	1	0	5	GTPase activity Anti‐viral defence IFNγ signalling
7	Lower in TB‐DM throughout; rising end treatment	10	4	1	0	5	Splice variant
8	Higher in TB‐DM throughout; decreasing with time	16	13	2	0	1	Secreted Neutrophil chemotaxis Transmembrane helix
9	Lower in TB‐DM at week 8 and 24, otherwise similar	4	3	0	0	1	Immunoglobulins

^a^ DAVID^49^ analysis of GO terms BP, MF, CC direct UP‐keywords.

^b^ Retained introns, antisense, LncRNA, miRNA, nonsense‐mediated decay, sense overlapping, sense intronic, snoRNA.

### 3.3 Aberrant longitudinal transcriptomes in TB patients with intermediate hyperglycaemia

Previously^27^ we showed that gene expression in TBrel‐IH is more similar to people with diagnosed DM and TB than with TB‐only at TB diagnosis. We repeated the MaSigPro analyses separately for South Africa and Indonesia, combining patients with pre‐DM and intermediate glycaemia, to determine how transcriptomes changed through TB treatment in intermediate groups (Figure S2). In South Africa, the analysis resulted in 1179 transcripts separated into three hierarchical clusters, which changed through treatment differently across clinical groups (Figure S2A and Table S5), with the combined intermediate group behaving more similarly to TB‐DM. Similar results were obtained with the Indonesian cohort, with 2354 transcripts across four hierarchical clusters behaving differently between clinical groups (Figure S2B and Table S6).

**FIGURE 4:**
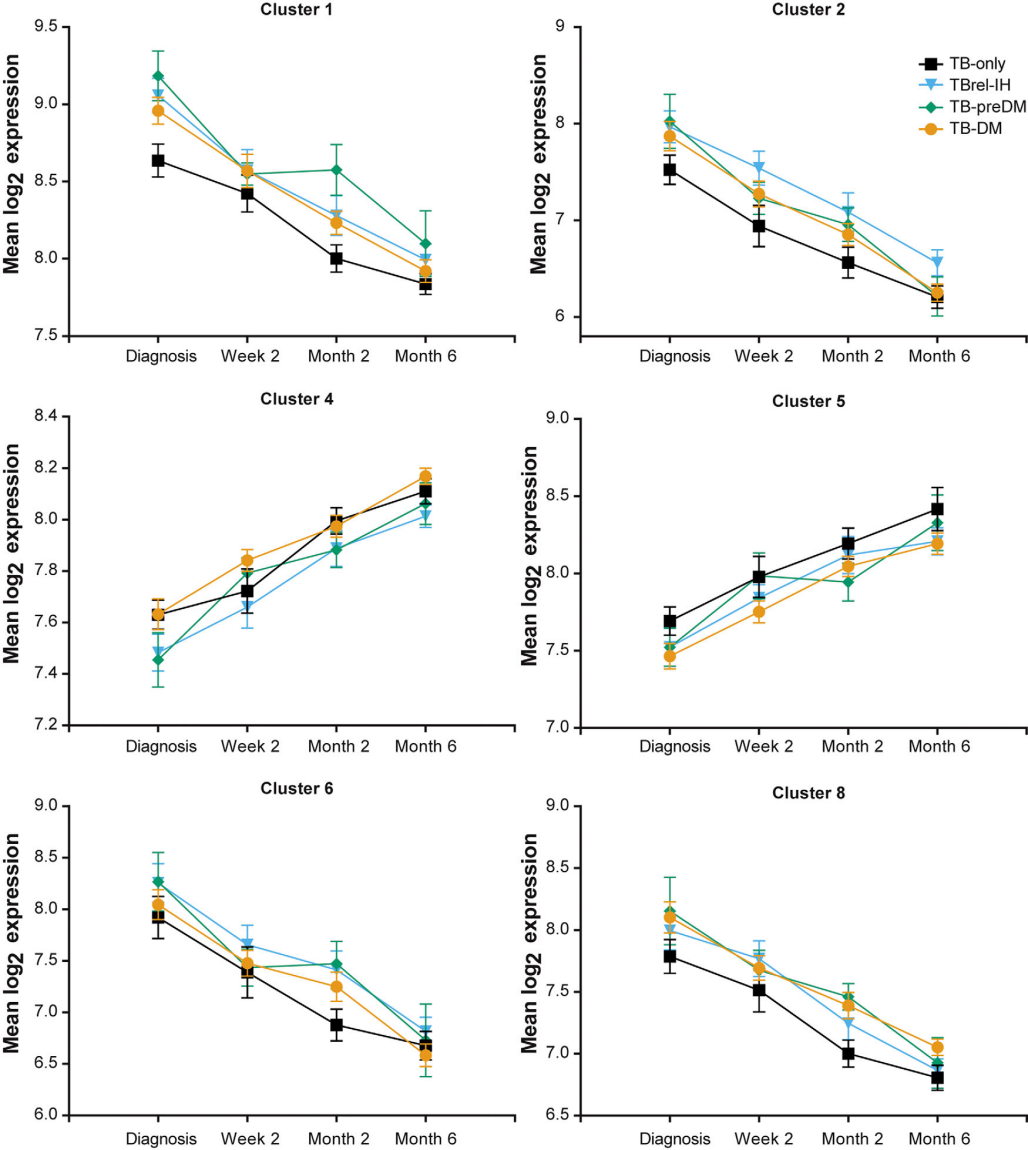
Gene expression through treatment in TB patients with pre‐diabetes or TB‐related intermediate hyperglycaemia, relative to TB‐DM and TB‐only patients. The expression of genes in the Core 102 genelist (Table S7) was summed for those genes within each MaSigPro gene cluster (Figure 3) for individual patients (log_2_ scale). Only MaSigPro clusters with >3 genes in the core gene list are shown. Points show the mean ± SEM for each of the four clinical groups at each timepoint.

A core list of 102 genes overlapped between MaSigPro analyses for the combined cohort from Romania, South Africa and Indonesia, and from the latter two populations separately (Figure S3 and Table S7). Gene ontology and pathway analyses of this core list using the g:profiler webtool revealed functional enrichment of genes involved in the immune response, in the response to biotic stimuli, and gene products localising to intracellular vesicles (Figure S4). We hypothesised there would be differences between the TB‐preDM and TBrel‐IH groups, as the initially elevated HbA1c in the TBrel‐IH group was directly ascribed to TB and resolved during TB treatment, whereas there was persistence of hyperglycaemia in the TB‐preDM group through TB treatment; however, there was no evidence to support this postulate, as gene expression changes in both TB‐preDM and TBrel‐IH patients were largely similar to each other, and the similarity to TB‐DM or TB‐only patient groups varied by gene cluster (Figure 4). Longitudinal mixed effects model analysis of mean expression within the core gene list clusters showed highly significant changes across all four clinical groups throughout treatment, with differences between the groups in larger gene clusters (Table S8). Importantly, there was no interaction between clinical group and time, showing there was resolution of expression in all groups through treatment, albeit from different starting points and at different rates.

### 3.4 Modular analysis of DEGs

**FIGURE 5:**
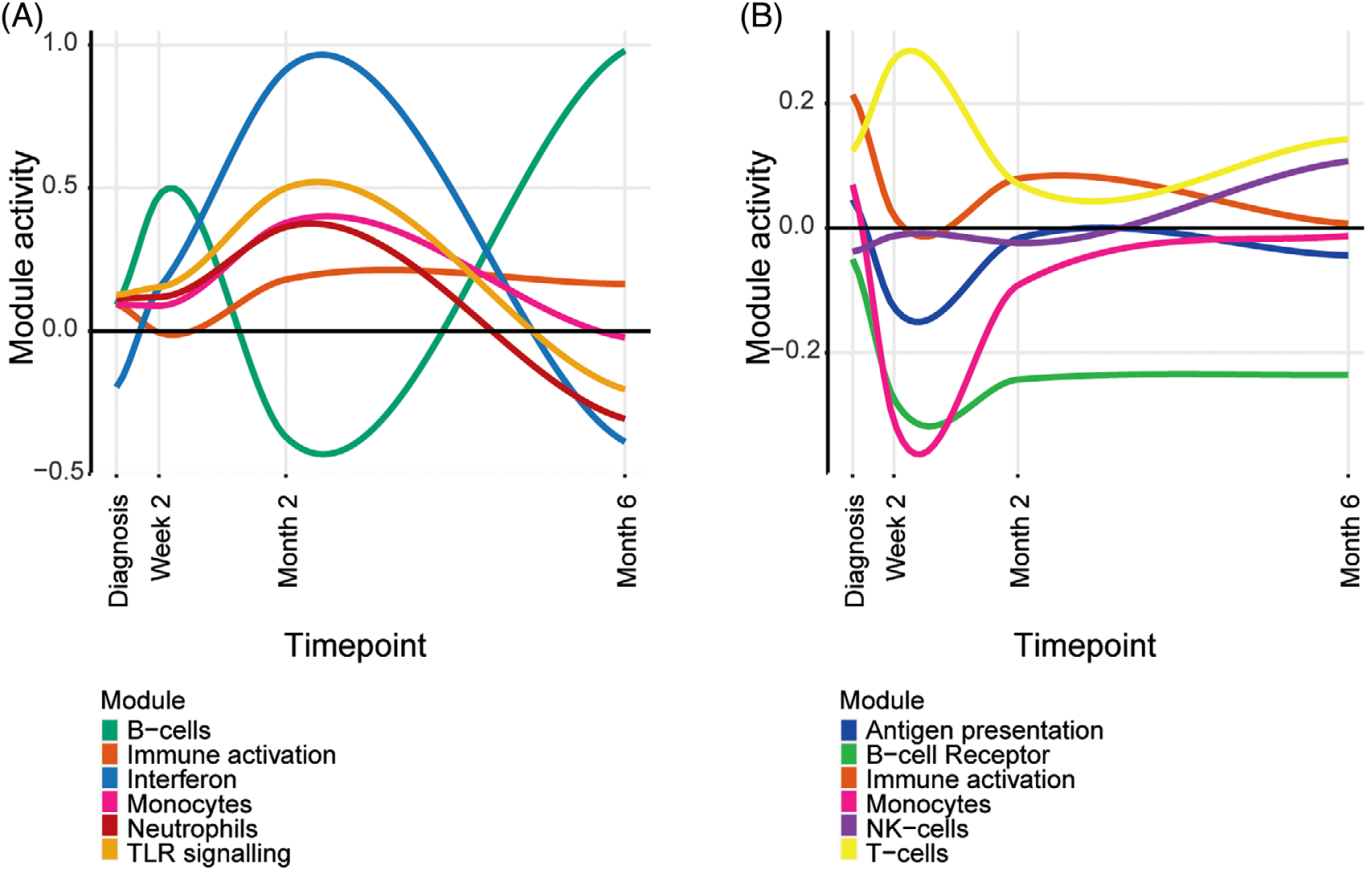
Modular activity of the most significant modules in TB‐DM relative to TB‐only in (A) South Africa and (B) Indonesia. Modular analysis was performed between TB‐DM and TB‐only patients and the most statistically significant were chosen (*p* value < .05). Modular activity calculated by summing the expression of genes within a module and dividing by the number of genes within that module.

DEGs identified in the MaSigPro analyses were used in modular analyses to understand biological differences between clinical groups in South Africa and Indonesia. The DEGs were used as a foreground signal against all genes (Tables S9 and S10 respectively). Immune activation, monocytes and neutrophils were the most statistically significant differentially expressed modules. The most statistically significant modules were investigated further by calculating their modular activity in TB‐DM relative to TB‐only through time. The top module in both populations was immune activation, which was upregulated in TB‐DM compared with TB‐only throughout treatment. In both populations, different modules fluctuated and behaved inversely to one another between TB‐DM and TB‐only (Figure 5).

### 3.5 Impact of DM on the TB treatment response using targeted gene expression profiling

**FIGURE 6:**
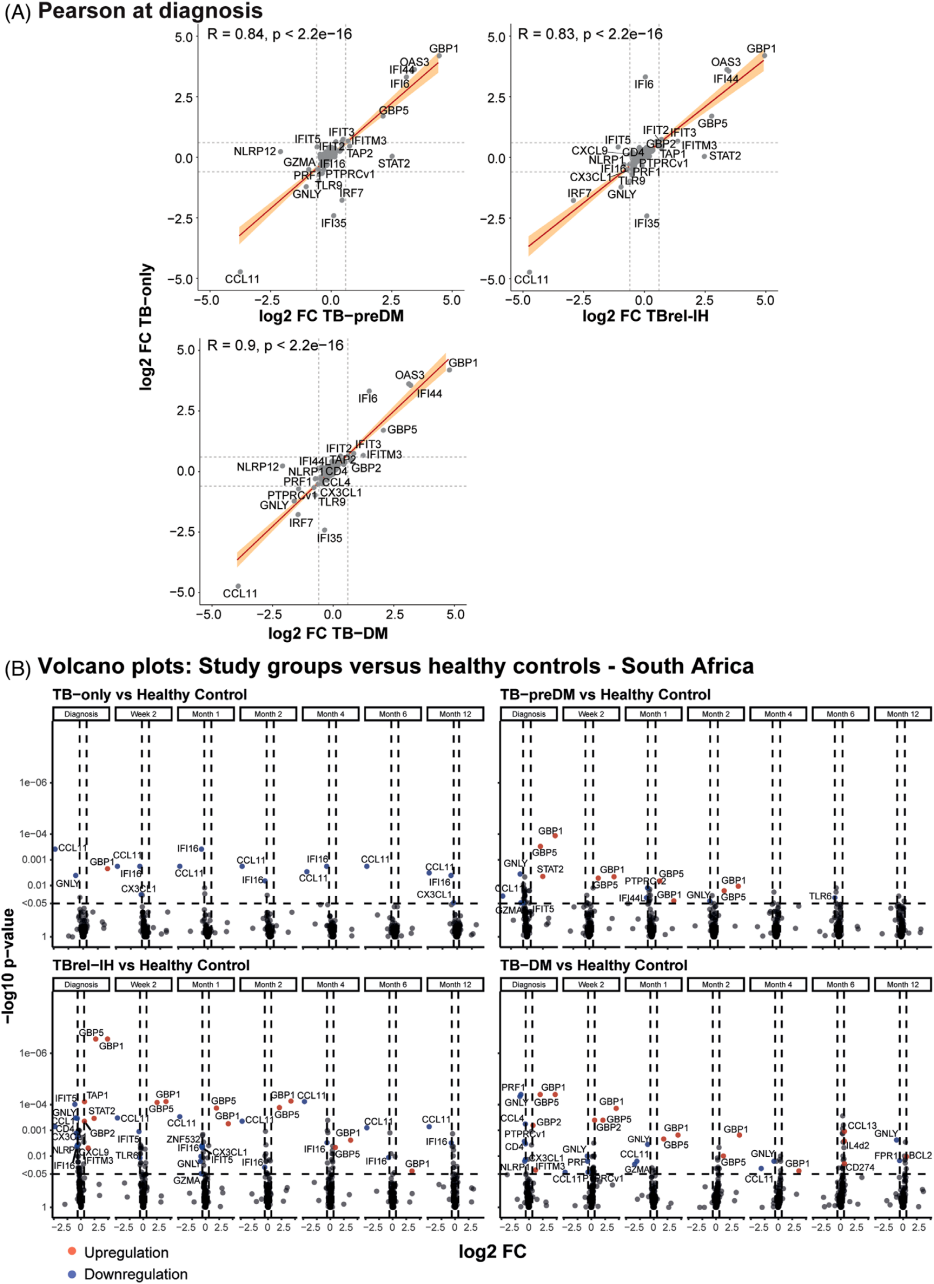
Gene expression profiles in TBrel‐IH and TB‐DM are not completely normalised to healthy control profiles at the end of TB treatment. (A) Scatter plots representing Pearson correlations between expression of all genes in targeted dcRT‐MLPA panel in TB patients relative to healthy controls (*y*‐axes) versus the other study groups relative to healthy controls (*x*‐axes), plotted as log_2_ FC. Red line corresponds to line of best fit and shaded bands indicate confidence intervals. Genes regulated log_2_ FC < ‐ .6 or > .6 are annotated. (B) Differential Expression Analysis was performed on GAPDH‐normalised log_2_‐transformed targeted gene expression data of the South African cohort. Volcano plots representing DEGs at diagnosis and at different timepoints post TB treatment initiation of TB patients categorised based on their diabetes/glycaemia status compared with the healthy controls. The *y*‐axis scales of all plots are harmonised per study group. *p* Values, ‐log_10_‐transformed for better visualisation, are plotted against log_2_ FC. Genes with *p* < .05 and log_2_ FC  < ‐.6 or > .6 were labelled as DEGs.

We performed targeted profiling of TB‐relevant immune gene expression in an expanded cohort from South Africa with more intensive sampling, using dcRT‐MLPA (TB‐DM: *n* = 19; TB‐PreDM: *n* = 28; TBrel‐IH: *n* = 32; TB‐only: *n* = 17; HC: *n* = 27; Table S11). At baseline, the overall gene expression perturbation, including genes from modules previously highlighted, was similar in all study groups and significantly increased compared with HCs (Figure S5A). PLS‐DA displayed a clear although partial separation of all the TB groups irrespective of DM or glycaemia from HCs, suggesting distinct genes are perturbed (Figure S5; B27). Gene expression was strongly correlated between TB‐only and TB‐DM, TB‐preDM or TBrel‐IH, but with some outlier genes which were affected by glycaemic status (Figure 6A). The number of DEGs relative to HCs was higher in the TB‐DM (*n* = 11 DEGs), TB‐preDM (*n* = 7 DEGs) and TBrel‐IH (*n* = 14 DEGs) groups than TB‐only (*n* = 3 DEGs) at TB diagnosis (Figure 6B). In these groups, the number of DEGs progressively reduced over time, indicating a resolution of expression through treatment. In particular, normalisation of expression of genes such as *GNLY* and *GBP1* occurred by 2 weeks in the TB‐only group but was delayed in TB‐DM, TB‐preDM and TBrel‐IH.

**FIGURE 7:**
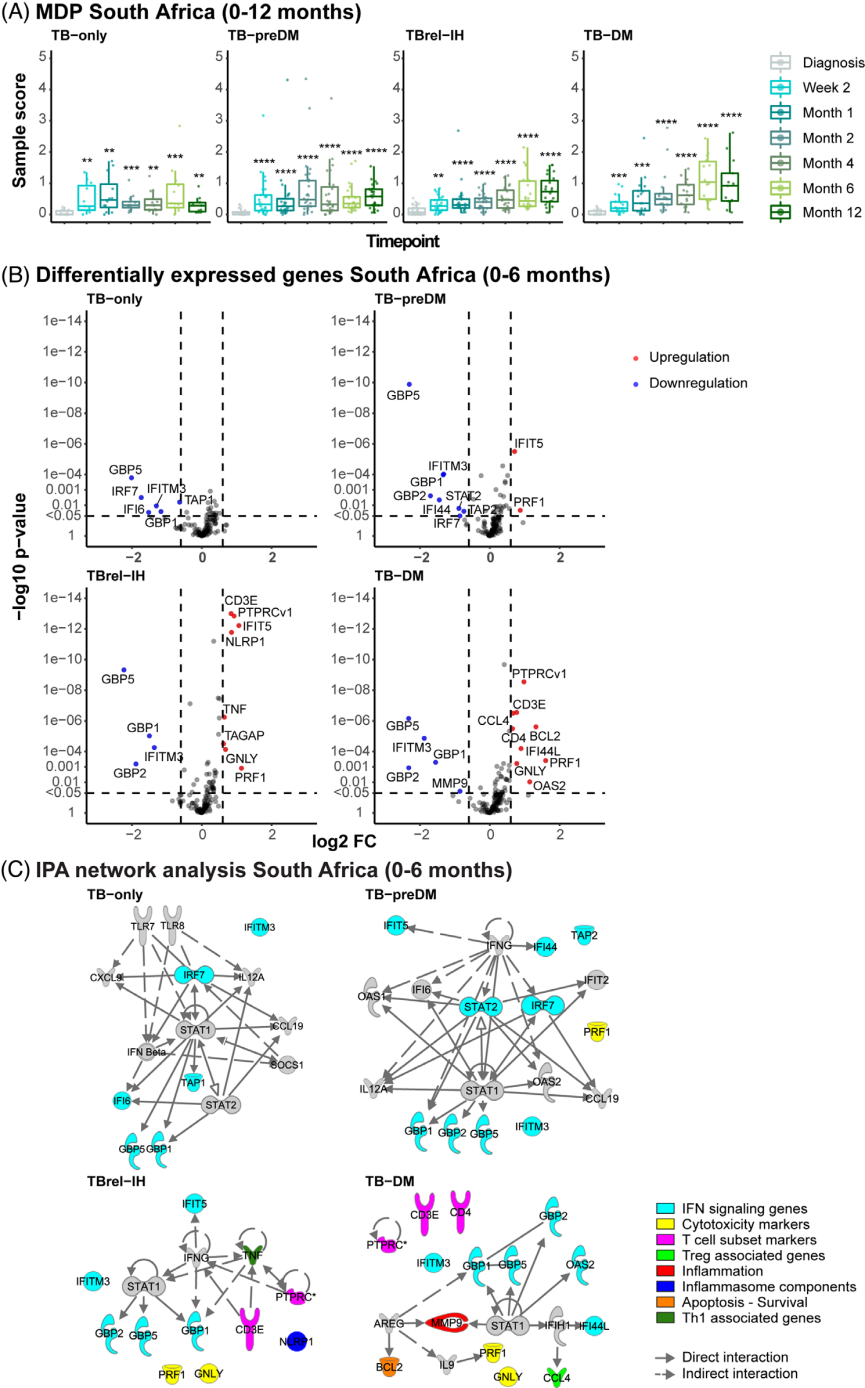
TB treatment response in TB patients is dependent on diabetes/glycaemia status. MDP and differential expression analyses were performed on GAPDH‐normalised log_2_‐transformed targeted gene expression data of the South African cohort. (A) MDP analyses of the different study groups showing the impact of TB treatment on the overall gene perturbation over time. Samples of patients at diagnosis were used as baseline controls. (B) Volcano plots representing DEGs regulated during TB treatment of TB patients categorised based on their diabetes/glycaemia status. The *y*‐axis scales of all plots are harmonised per study group. *p* Values, ‐log_10_‐transformed for better visualisation, are plotted against log_2_ FC. Genes with *p* < .05 and log_2_ FC  < ‐.6 or > .6 were labelled as DEGs. (C) IPA interactive network analyses of DEGs regulated during TB treatment. The various shapes of the nodes represent the functional classes of the gene products. Gene modules are indicated by distinctive colours.

Longitudinal MDP analysis in the South African (TB‐DM: *n* = 19; TB‐PreDM: *n* = 28; TBrel‐IH: *n* = 32; TB‐only: *n* = 17) and Indonesian cohorts (TB‐DM: *n* = 41; TB‐PreDM: *n* = 0; TBrel‐IH: *n* = 19; TB‐only: *n* = 20) indicated the magnitude of the transcriptomic response to TB treatment was dependent on diabetes/glycaemia, with TB‐DM patients displaying the largest gene expression perturbation over time (Figures 7A and S6A). Gene expression changes through treatment, identified by linear mixed models, showed some consistency across TB groups, with the South African cohort exhibiting downregulation of *GBP5*, *GBP1* and *IFITM3* (Figure 7B) and the Indonesian cohort showing downregulation of *GBP5* and *IFITM3* and upregulation of *GNLY* (Figure S6B) from diagnosis to 6 months. Importantly, the number of upregulated DEGs in response to TB treatment increased with rising glycaemia in both cohorts (South Africa: TB‐only: 6 DEGs, TB‐preDM: 10 DEGs, TBrel‐IH: 12 DEGs, TB‐DM: 14 DEGs; Indonesia: TB‐only: 9 DEGs, TBrel‐IH: 13 DEGs, TB‐DM: 22 DEGs). We did not find any evidence that the change in the expression of the most significantly DEGs correlated with the change in the glycaemic control in the TB‐DM group, tested in the Indonesian cohort (Figure S7), suggesting this is an independent measure of TB disease resolution. Notably, no DEGs were detected between 6 and 12 months in the South African cohort, except for *GBP5* (*p* < 1e−10) in patients with TBrel‐IH (Figure S8).

**FIGURE 8:**
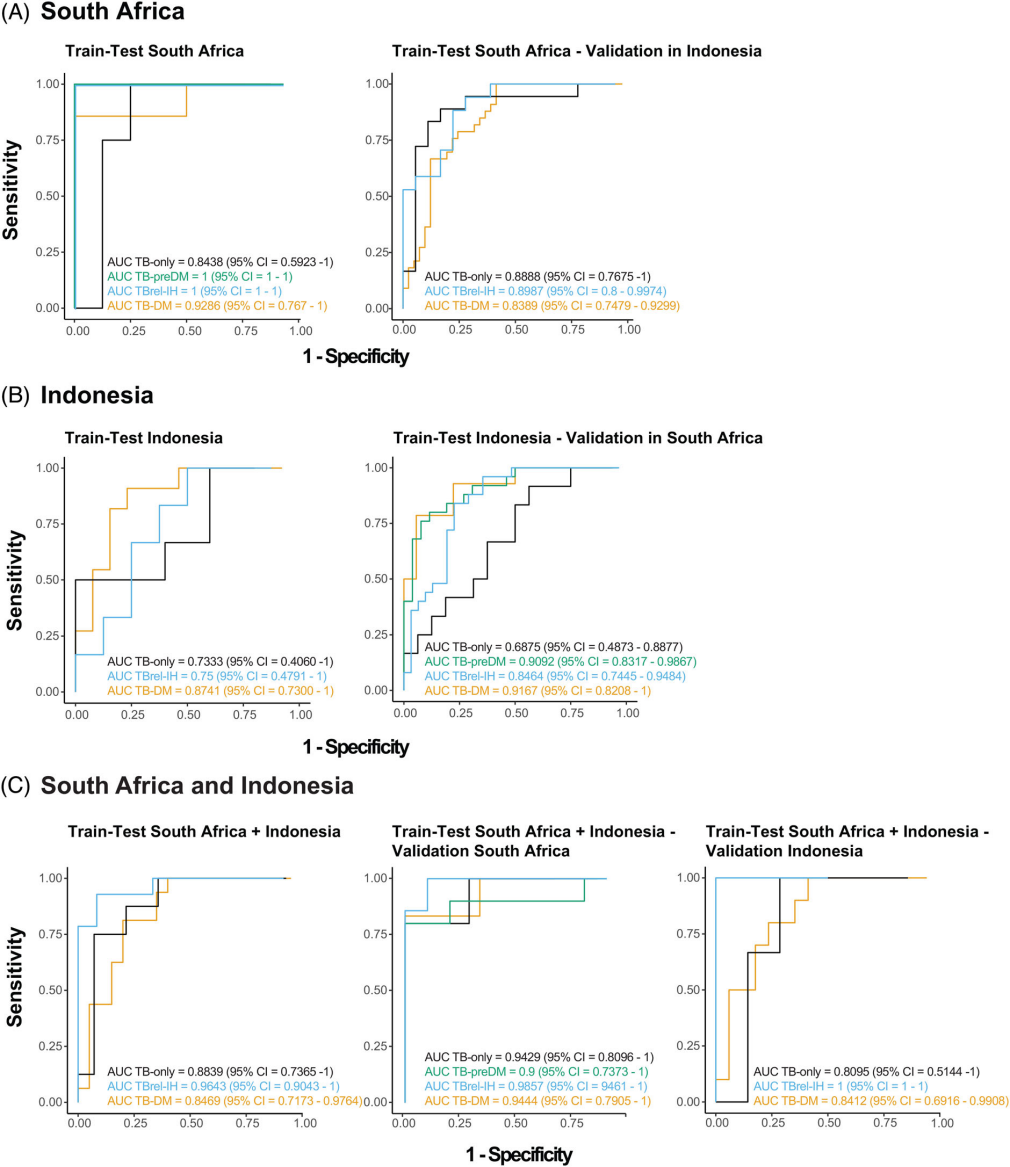
Identification of common host biomarker signatures associated with TB treatment response irrespective of population heterogeneity and diabetes/glycaemia severity. South African, Indonesian or pooled cohort transcriptomic datasets of TB patients independent of their diabetes/glycaemia status were used to train the models. Receiver operating characteristic (ROC) curves (sensitivity plotted against 1‐specificity) and area under the curve (AUC) with 95% confidence intervals (CI) show the classifying performance of the trained models. (A) The model trained on 70% of the South African dataset was tested in the remaining 30% of the South African dataset split into the different TB study groups (left panel) and validated using the complete dataset of the Indonesian cohort split into the different TB study groups (right panel). (B) The model trained on 70% of the Indonesian dataset was tested in the remaining 30% of the Indonesian dataset split into the different TB study groups (left panel) and validated using the complete dataset of the South African cohort split into the different TB study groups (right panel). (C) The model trained on 70% of the pooled (South African and Indonesian) dataset was tested in the remaining 30% of the pooled dataset split into the different TB study groups that both cohorts have in common (left panel) and validated using the complete dataset of the South African cohort split into the different TB study groups (middle panel) or the complete dataset of the Indonesian cohort split into the different TB study groups (right panel).

Ingenuity pathway analysis showed the majority of treatment‐response DEGs in TB‐only and TB‐preDM were IFN‐signalling genes (ISGs) (Figures 8C and S6C). In contrast, in TBrel‐IH and TB‐DM patients, although downregulation of ISGs through treatment was observed, the major change was upregulation of genes associated with adaptive immunity (T‐cell subset markers, Th1‐associated genes, Treg‐associated genes, cytotoxicity markers). Overall, the dcRT‐MLPA confirmed that although TB‐associated gene profiles showed similar patterns and rate of change in TB patients and people with TB‐DM, the magnitude was different.

### 3.6 Identification of a signature for TB treatment‐response

As TB transcriptomic signatures were altered in people with DM or IH, we identified signatures with the highest classifying power to discriminate between patients at diagnosis and end of TB treatment irrespective of diabetes/glycaemia by pooling all TB patients, using logistic regression with lasso regularisation. Initially, signatures were developed in the South African and Indonesian cohorts separately (Tables 2 and S12). The classifying capability of each signature against the training (AUC range: 0.73–1.0) and validation (AUC range: .69 – .92) cohorts for each clinical group was reasonably good (Figures 8A and B and S9A and B). To improve the classification performance and reduce cohort dependency, the datasets of both cohorts were pooled, and a combined two cohort 15‐gene signature developed. This showed enhanced classification performance across the cohorts, with ROC analysis showing AUCs of .88 for TB‐only, .96 for TBrel‐IH and .85 for TB‐DM, with excellent classification retained in individual cohorts (Figures 8C and S9C). The kinetic profiles of six representative genes are shown in Figure S10.

**TABLE 2:** Gene expression signature predicting month 6 versus diagnosis, obtained by pooling the study groups and cohorts (South Africa + Indonesia).

Gene	Module	Coefficient
Intercept		−14.99967
BLR1	G protein‐coupled receptors	.07841
CCL13	Chemokines	.40415
CCL4	Treg‐associated genes	.66015
CD19	Immune cell subset markers—B cells	.2371
CD3E	T cell subset markers	.35071
CD4	T cell subset markers	.00263
FCGR1A	IFN signalling genes	−.48224
FPR1	Myeloid‐associated genes	−.1725
GBP5	IFN signalling genes	−.16957
IFIT5	IFN signalling genes	.0172
NLRP1	Inflammasome components	.3498
PTPRCv1	T cell subset markers	.6625
TAP1	IFN signalling genes	−.51107
TNF	Th1‐associated genes	.06539
ZNF532	Transcriptional regulators/activators	.06389

Ingenuity pathway analysis revealed the top network of genes included in the model were centred on TNF/NF‐κB/MAPK (Figure S11A), with the top upstream regulators including proteins such as natural cytotoxicity triggering receptor and UL16 binding protein, which are involved in NK cell mediated killing, as well as the binding partners solute carrier family 15 member 4 and TLR adaptor interacting with endolysosomal SLC15A4, which are involved in regulation of TLR7 and TLR8 signalling (Table S13). An online search revealed limited drugs currently available or under development to target these regulators. This analysis was extended to include the genes from the RNASeq global MaSigPro which were upregulated in TB, downregulated by TB treatment but consistently more highly expressed in TB‐DM, that is, genes in Clusters 1, 2, 6 and 8 from Figure 3. The top network identified was an inflammatory network centred on NF‐κB and MAPK, alongside the IFN response (Figure S11B). The top canonical pathways identified included inflammasomes and cytokine signalling. Various compounds exist which target some of the upstream regulators identified, such as Emapalumab for IFNγ, anakinra for IL‐1α, or H‐151 which is under development for STING1 antagonism (Table S13).

## 4 DISCUSSION

In this longitudinal analysis of blood transcriptomes, excessive gene expression perturbation previously described at TB diagnosis^14^, ^27^ continued throughout six months of TB treatment in pulmonary TB patients with diabetes co‐morbidity. However, qualitatively and kinetically similar changes occurred in patients with or without diabetes, suggesting prolonged TB treatment might be sufficient to restore normal transcriptomes and potentially improve TB treatment outcomes: this would need to be tested in a clinical trial. Whilst DM itself causes altered blood transcriptomes, we have previously shown these are qualitatively different to the changes seen in TB‐DM,^27^ making it unlikely the remaining TB‐related transcriptomic signature in TB‐DM patients is caused by DM directly. TB patients with either pre‐diabetes or TB‐related IH also exhibited greater magnitudes of gene expression perturbation throughout treatment, similar to patients with diagnosed diabetes. There was no clear difference between the pre‐diabetes and TB‐related IH groups through treatment, indicating that aberrant glycaemic control in TB and early in TB treatment is sufficient to cause prolonged excessive gene expression abnormalities despite resolution of glycaemic control in the latter group. Our data published here and in our previous cross‐sectional study^27^ are not fully consistent with results reported by Prada‐Medina *et al* ^13^ in an Indian TB‐DM cohort, whereby they concluded that the differences in the TB‐DM transcriptome compared with TB‐only were largely driven by diabetes‐related signatures: the difference may be related to the classification of participants as we have only included people with no evidence of any hyperglycaemia in our clinical group. The patient classification utilised in this study was primarily based on measures of hyperglycaemia. People with type 2 diabetes have complex metabolic and lipid disturbances, including elevated triglycerides, reduced high‐density lipoproteins and increased low‐density lipoproteins in blood. Such changes have also been observed in TB‐DM co‐morbidity,^50^ with measurements of altered carbohydrate, amino acid and lipid metabolism able to clearly discriminate between TB and TB‐DM.^51^ It is likely not the hyperglycaemia per se which has caused the alterations in the gene expression in people with TB‐DM in the current study: rather this would be the overall result of the complex metabolic disturbance. Here, the overall consistency in change of gene expression through treatment, irrespective of diabetes status, enabled derivation of accurate predictive models of TB treatment response, which could be used effectively in populations with or without diabetes.

Diabetes has a negative effect on TB treatment outcomes,^5^, ^6^ for unclear reasons. One explanation could be a qualitatively different immune response in diabetes, leaving people persistently susceptible to bacterial replication and disease reactivation. An alternative explanation is that excessive inflammation and immune activation at diagnosis in TB‐DM means patients require longer or, more likely, different treatment to reach the same endpoint as people with uncomplicated TB, so that they are not left susceptible to TB recurrence. Our data support the latter model, as all gene clusters differentially expressed between clinical groups exhibited similar changes, but of different magnitude. Prolonged enhanced concentrations of the pro‐inflammatory cytokines IL‐1β and TNFα in blood, and reduced anti‐inflammatory IL‐10 in sputum, have been observed in patients with TB and diabetes comorbidity,^52^ in accordance with our results. Bronchial spread often persists beyond treatment initiation, with new or expanding cavities appearing on PET‐CT scans 4 weeks into treatment in one‐fifth of pulmonary TB patients.^53^ Plausibly, increased ongoing bacterial spread in patients with diabetes co‐morbidity causes persistent pro‐inflammatory responses: the peripheral transcriptome correlates with lung inflammatory activity in TB patients.^54^ Restoration of normal transcriptomes, and presumably improved lung resolution, could potentially also be achieved by co‐administration of host‐directed therapy alongside standard treatment. Therapy which dampens pro‐inflammatory responses, such as corticosteroids or matrix metalloproteinase inhibitors,^55^ would have added benefit by reducing lung damage, which often persists after microbiological cure.^56^ Our data suggest upstream regulators of inflammatory responses, such as inhibition of IL‐1α or STING1, might provide tractable drug targets for TB‐DM comorbidity. The role of specific DEG or of upstream regulators could be tested using mouse models of diabetes coupled with CRISPR technology, to determine the impact on TB disease pathology and treatment response following infection with *M. tuberculosis*. Anti‐hyperglycaemic therapy, such as metformin, leads to more balanced, less inflammatory responses to *M. tuberculosis*,^57^ and has been suggested as adjunctive therapy for TB, particularly in patients with diabetes.^58^ Our transcriptomic data suggest that patients with either pre‐diabetes or TB‐related IH would also benefit from prolonged or adjunctive host‐directed therapy, in alignment with the observed worse TB treatment outcomes in people with transient hyperglycaemia.^59^ Improved DM management per se might also improve TB treatment outcomes, but there is currently weak evidence in this field and larger clinical trials are warranted worldwide.^60^ In one recent study in the UK, TB patients with well‐controlled DM appear to have had normal TB treatment outcomes,^61^ whereas we have previously found that many TB patients with DM have been very poorly managed across four high TB burden countries.^29^

The ability to monitor TB treatment and predict outcome would be beneficial for clinical management. We show that transcriptomic models can be derived from host blood which reflect TB treatment‐response irrespective of glycaemia. These models worked well across geographically and ethnically diverse populations, enhancing their utility for drug development. There were however substantial differences between the three populations in Indonesia, South Africa and Romania, potentially reflecting different genetics of both host and pathogen, alongside other parameters such as social determinants including smoking and alcohol consumption, and exposure to other microbes. The best models include genes involved in IFN signalling, known to be suppressed at TB diagnosis in TB‐DM patients,^27^ which we found were enhanced mid‐way through treatment but did eventually resolve by 6 months. People protected against TB development display balanced prostaglandin E2 and lipoxin expression in lungs, preventing TB disease progression following infection.^62^ Drugs which target 5‐lipoxygenase restrict lung pathology and reduce bacterial replication in murine models, by lowering the type 1 IFN response^63^; the increases through treatment in the TB‐DM cohort may plausibly relate to sustained infection and accompanying inflammation. In TB‐DM patients, the inflammation‐related genes resolved more linearly through TB treatment, but remained elevated to the end of TB treatment, persisting until 12 months post‐diagnosis in the South African cohort. In future studies, it would be important to test whether prolonged treatment with standard therapy impacts blood transcriptomes beyond the 6 month time point. Increased doses of anti‐TB drugs might also lead to better treatment outcomes in TB‐DM. In a complementary paper,^26^ transcriptomic signatures indicative of treatment outcome have been derived that can be used in patients with either DM or IH. Together, these papers show that signatures related to poor TB outcome are distinct from the excessive and prolonged inflammation observed in TB‐DM. A strength of our study is the inclusion of several timepoints through TB treatment, particularly in the South African cohort, allowing a detailed kinetic analysis of how gene expression resolves in people with TB‐DM. Ahead of the study, we did not know whether gene expression resolution would be similar between people with TB‐DM and TB but of a different magnitude, whether there would be delayed kinetic of expression resolution, or whether there would be qualitatively different changes in gene expression – all of these scenarios would have been in keeping with the increased poor treatment outcomes experienced by people with TB‐DM. Theoretically, transcriptomic differences between people with TB‐DM and people with TB‐only might be caused by diabetes medications; however, we consider this to be unlikely as we have shown that people with TB‐IH, who are not taking any diabetes medicines, have similar profiles to those with TB‐DM who are.^27^ Also, the administration of the diabetes drug metformin, which is widely used by people with TB‐DM in this study, has no effect on ex vivo blood transcriptomes when administered to healthy individuals.^57^

The strengths of our study include the detailed clinical and temporal characterisation of TB patients with or without diabetes from three cohorts from three continents, with varied genetic and social backgrounds, with the derivation of a biosignature of TB treatment which applies across all groups. The limitations of the current study include the modest sample size, and that not all samples were analysed in depth by RNASeq to quantify the entire transcriptome: our data support the undertaking of a large scale prospective clinical study of biosignatures for the prediction of delayed immune/inflammatory resolution in TB and diabetes comorbid patients. Such a study should also include HCs and people with diabetes only in all cohorts, with samples collected longitudinally from these groups: this was another limitation in the current study, as samples were only available from these groups at one time point and only in South Africa and Romania. In this study, we used HbA1c to characterise people with TBrel‐IH and pre‐diabetes. A more comprehensive approach would have included impaired fasting glucose and impaired oral glucose tolerance test; however, these markers overlap and all are associated with future risk of diabetes.^64^ People with diabetes often have a range of clinical complications, such as heart disease, kidney disease, nerve damage and problems with other infections. Our study was not powered to investigate the impact of diabetic complications on gene expression as only two TB‐DM participants in South Africa and two in Indonesia had experienced significant clinical complications. A future biomarker large scale study should include assessment of the impact of these potential confounders on the TB‐DM transcriptome, which was beyond the scope of the current study. It would also be valuable to follow up people with pre‐diabetes and who are ‛latently’ infected with *M. tuberculosis*, to determine how these conditions drive each other long‐term.

These findings further illustrate how comorbidity with diabetes affects the host response to *M. tuberculosis* infection and to antibiotic treatment, and how a better understanding of these interactions could be exploited to reduce poor TB treatment outcomes associated with TB and diabetes comorbidity.

## CONFLICT OF INTEREST STATEMENT

G. W. holds patents about methods of tuberculosis diagnosis and tuberculosis biomarkers which are unrelated to the current study. No other authors have any declared conflicts of interest.

## FUNDING INFORMATION

The research leading to these results, as part of the TANDEM Consortium, has received funding from the European Community's Seventh Framework Programme (FP7/2007‐2013) under Grant Agreement no. 305279. THMO also received funding from the Netherlands Organization for Scientific Research (NWO‐TOP Grant Agreement No. 91214038).

## Supporting information

Supporting Information

Supporting Information

Supporting Information

Supporting Information

Supporting Information

Supporting Information

Supporting Information

Supporting Information

Supporting Information

Supporting Information

Supporting Information

Supporting Information

Supporting Information

Supporting Information

Supporting Information

Supporting Information

Supporting Information

Supporting Information

Supporting Information

Supporting Information

Supporting Information

Supporting Information

Supporting Information

Supporting Information

## Data Availability

The data that support the RNA‐Seq findings of this study are openly available in NCBI‐GEO at https://www.ncbi.nlm.nih.gov/geo/, accession number GSE193978. The data that support the dcRT‐MLPA findings of this study are available in the supplementary material of this article.
