# Supplementary figures and images for: Impaired resolution of blood transcriptomes through tuberculosis treatment with diabetes comorbidity
Source: Clin Transl Med. 2023 Aug 30;13(9):e1375. doi: 10.1002/ctm2.1375 (PMC10468587; doi:10.1002/ctm2.1375)

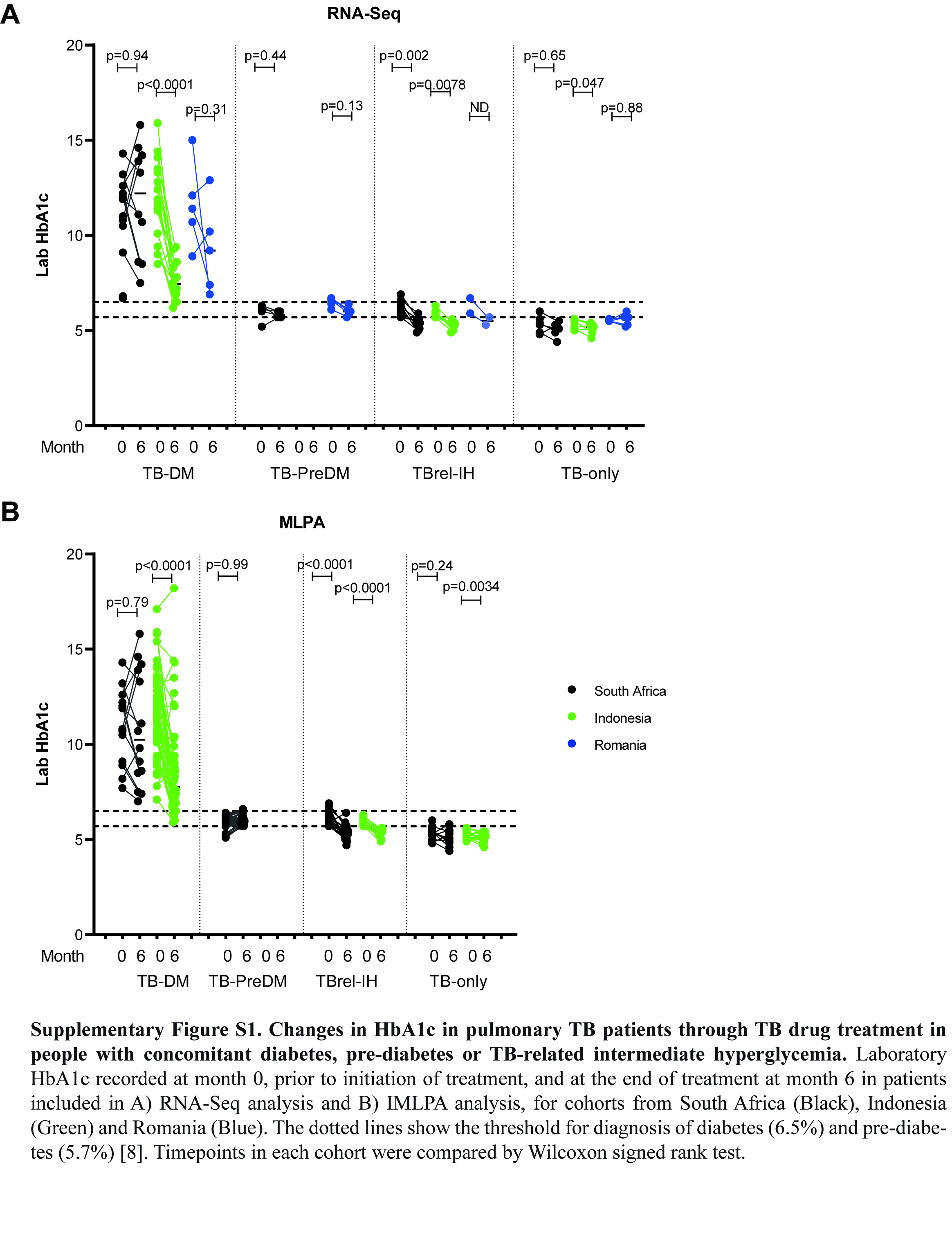

Supplement: Supplementary file 1 — Supporting Information [file CTM2-13-e1375-s019.tif]

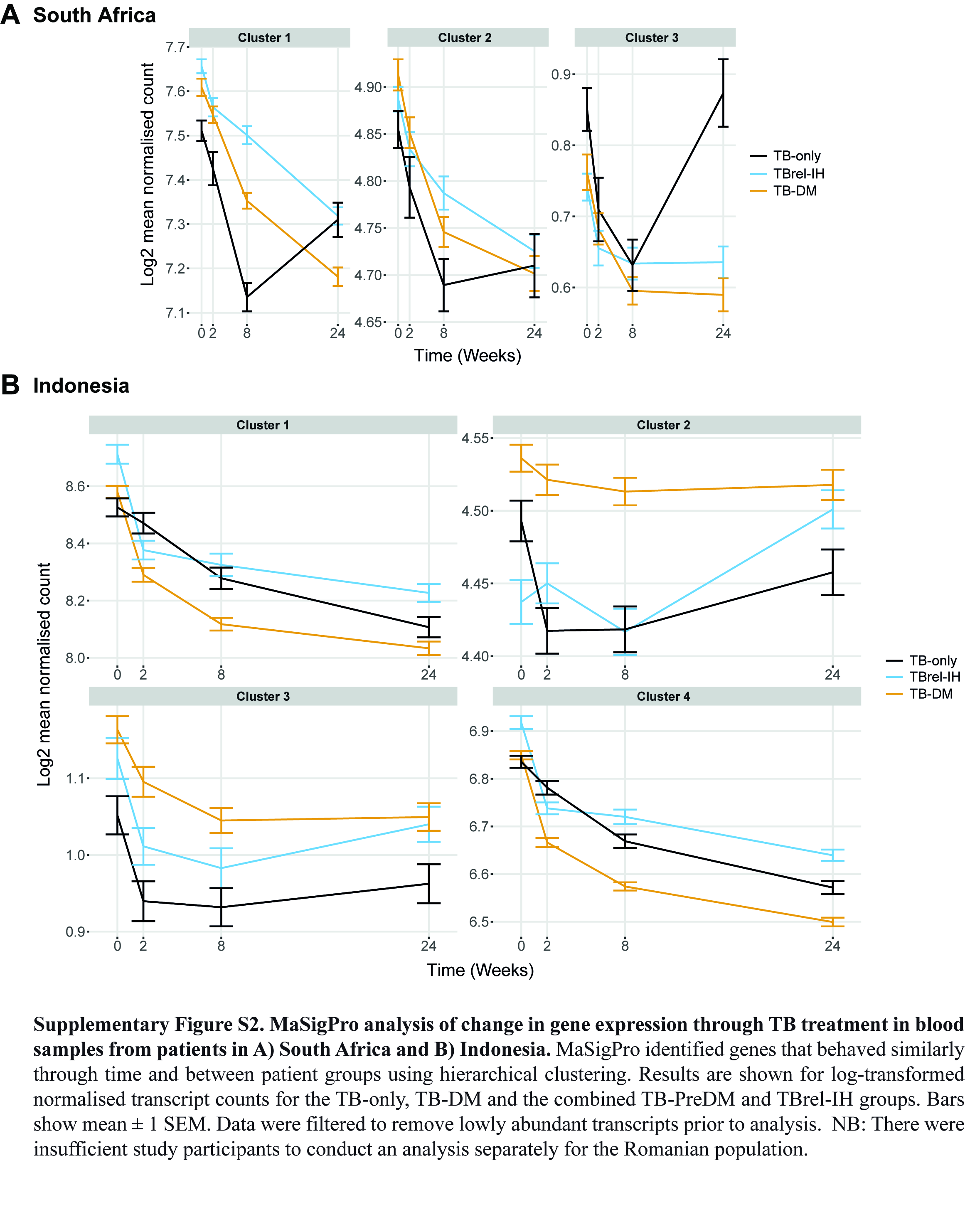

Supplement: Supplementary file 2 — Supporting Information [file CTM2-13-e1375-s020.tif]

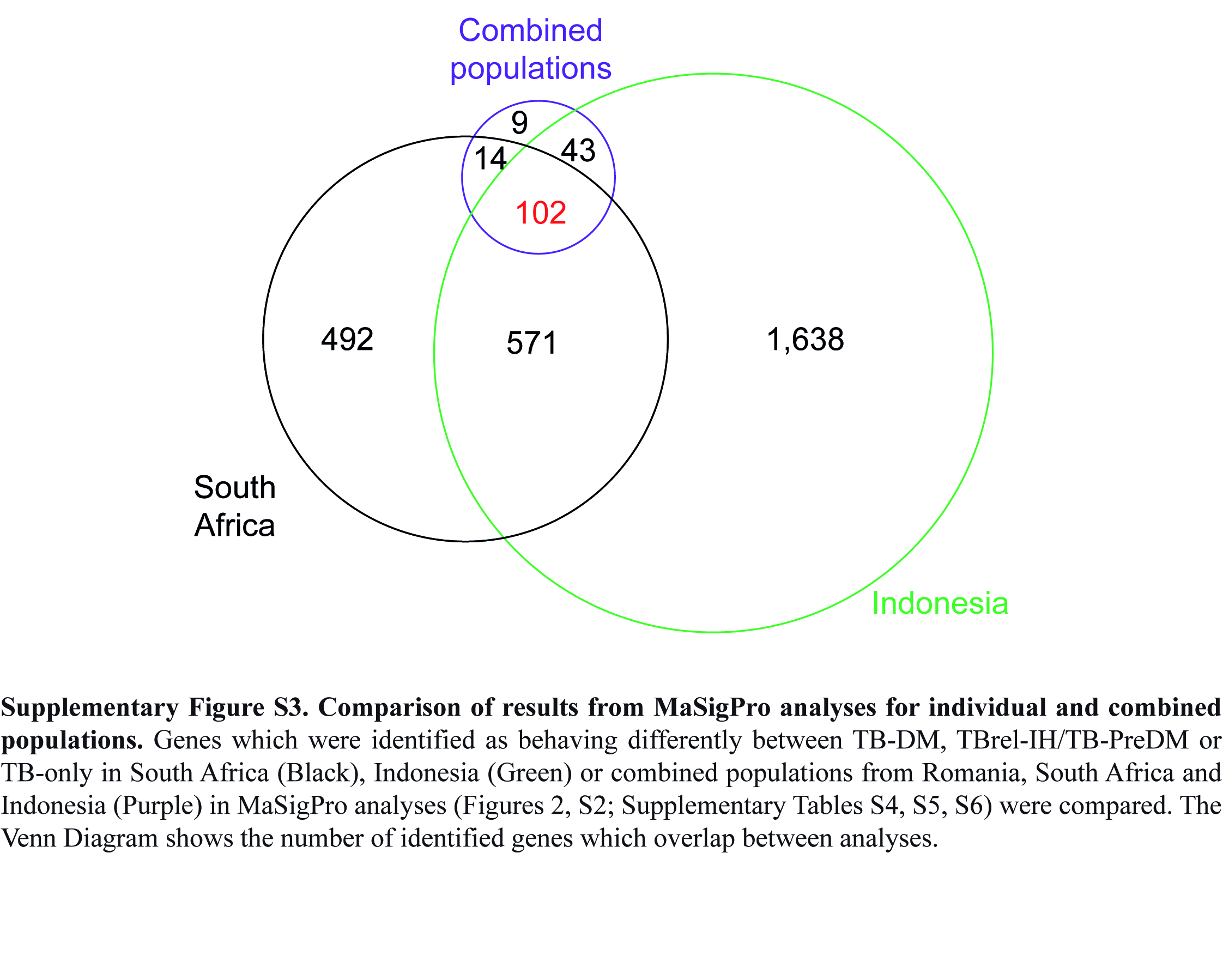

Supplement: Supplementary file 3 — Supporting Information [file CTM2-13-e1375-s007.tif]

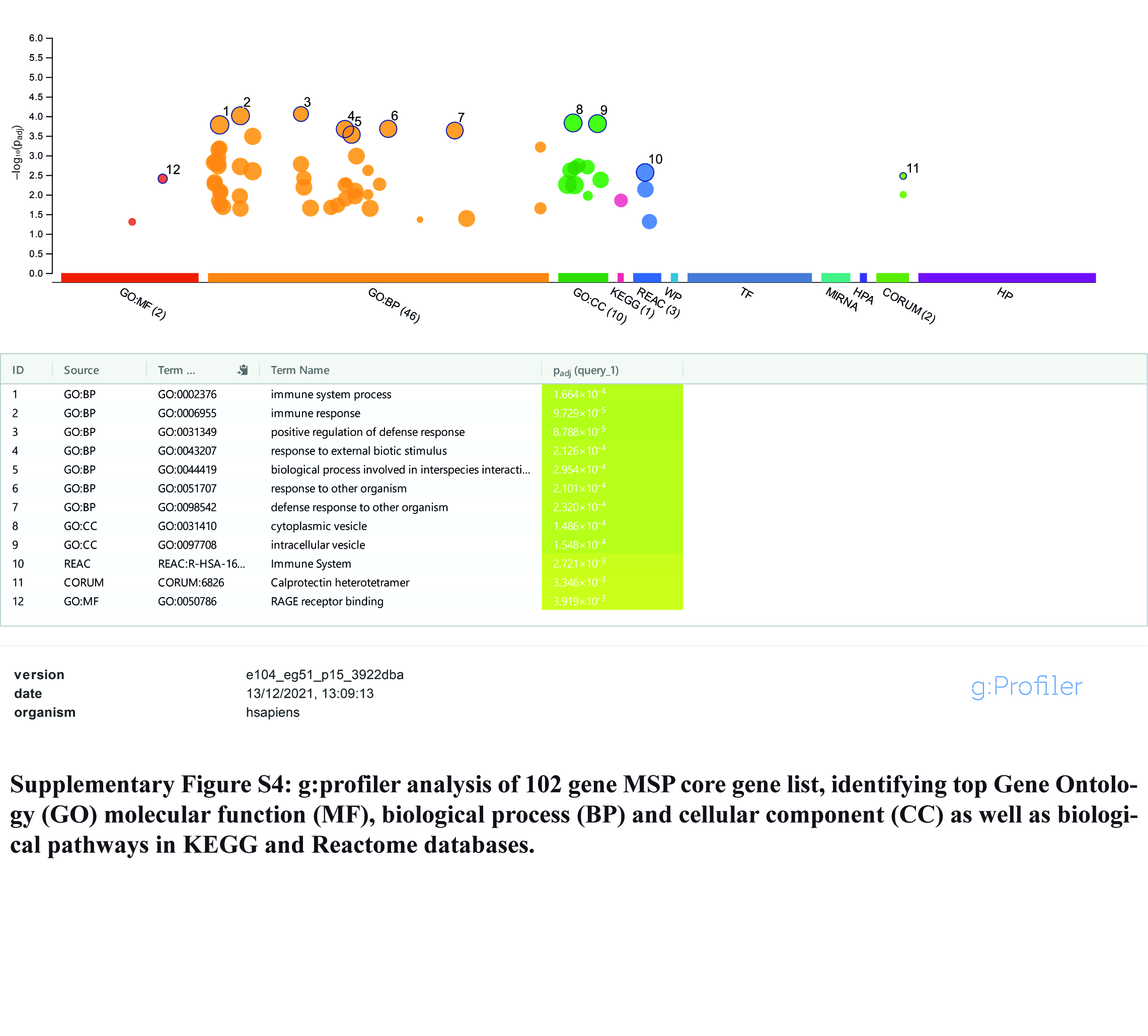

Supplement: Supplementary file 4 — Supporting Information [file CTM2-13-e1375-s013.tif]

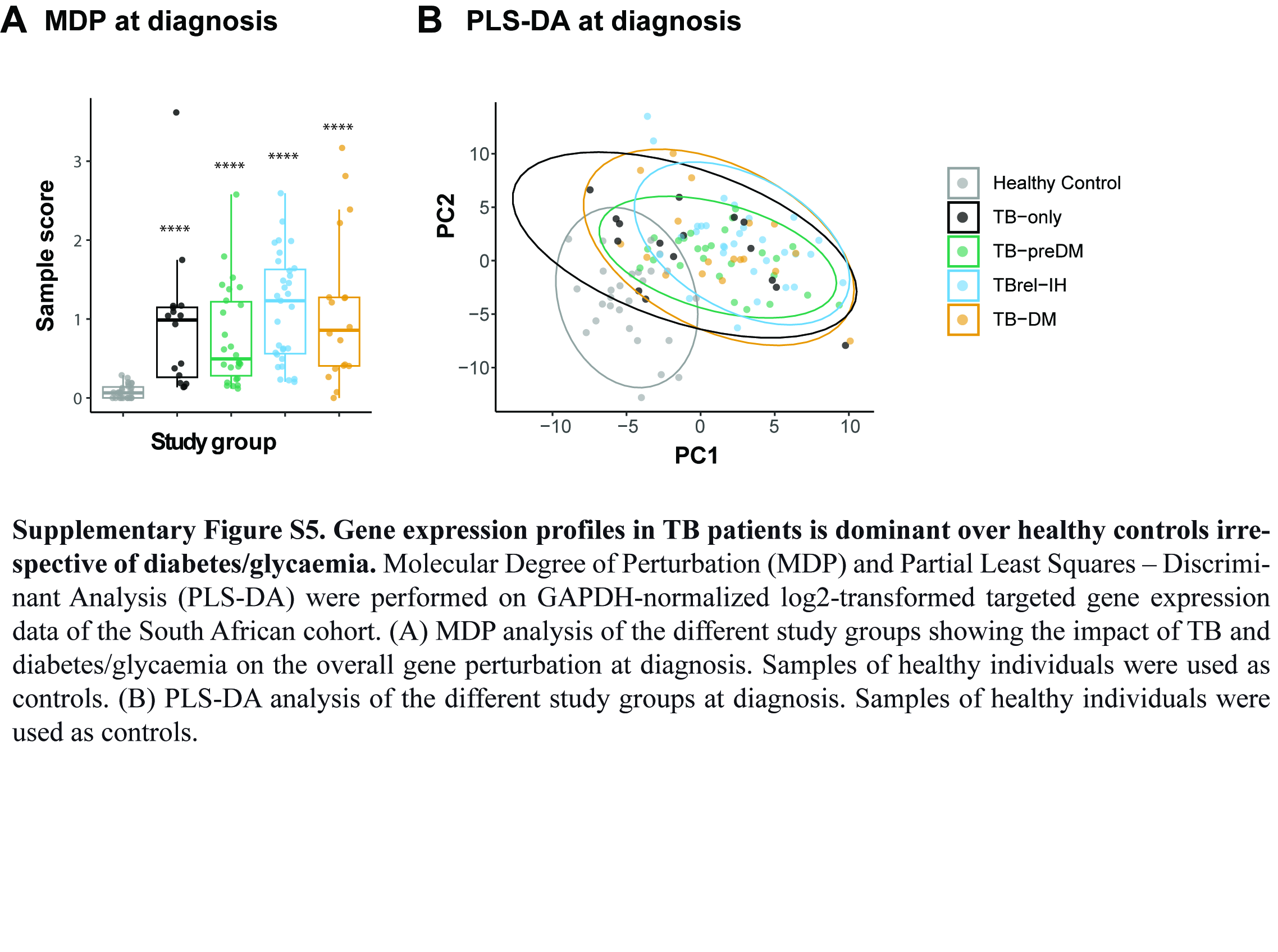

Supplement: Supplementary file 5 — Supporting Information [file CTM2-13-e1375-s005.tif]

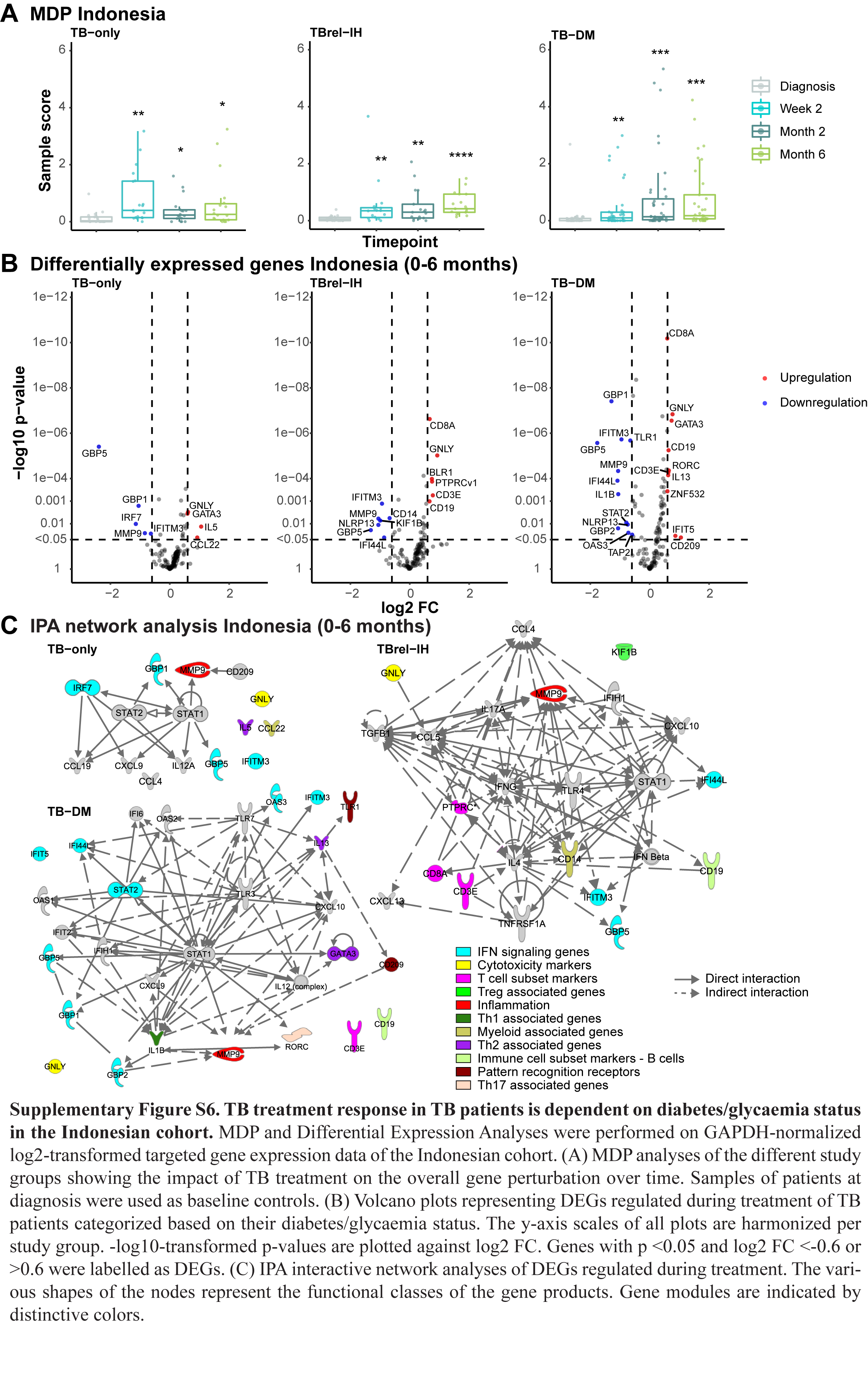

Supplement: Supplementary file 6 — Supporting Information [file CTM2-13-e1375-s023.tif]

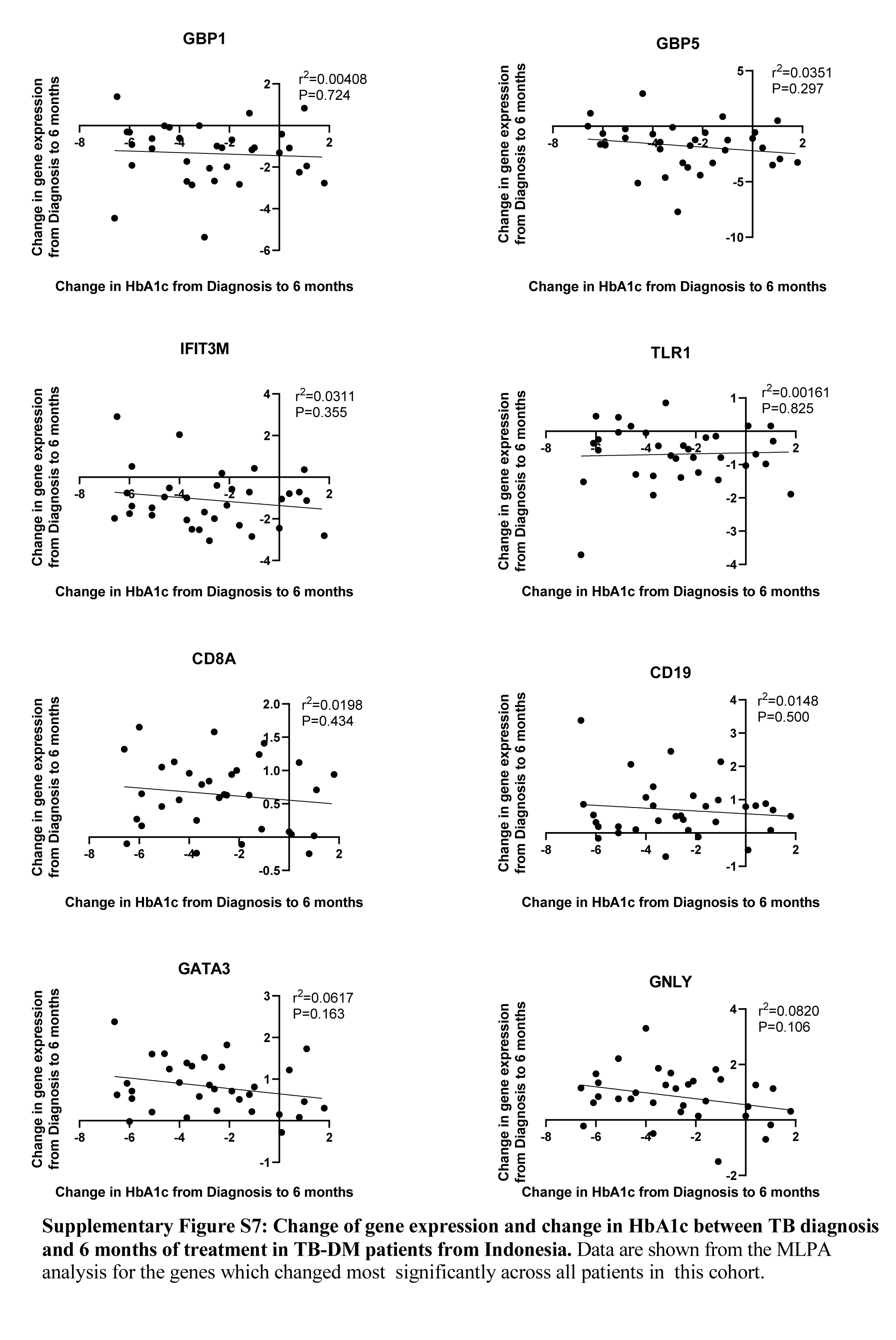

Supplement: Supplementary file 7 — Supporting Information [file CTM2-13-e1375-s015.tif]

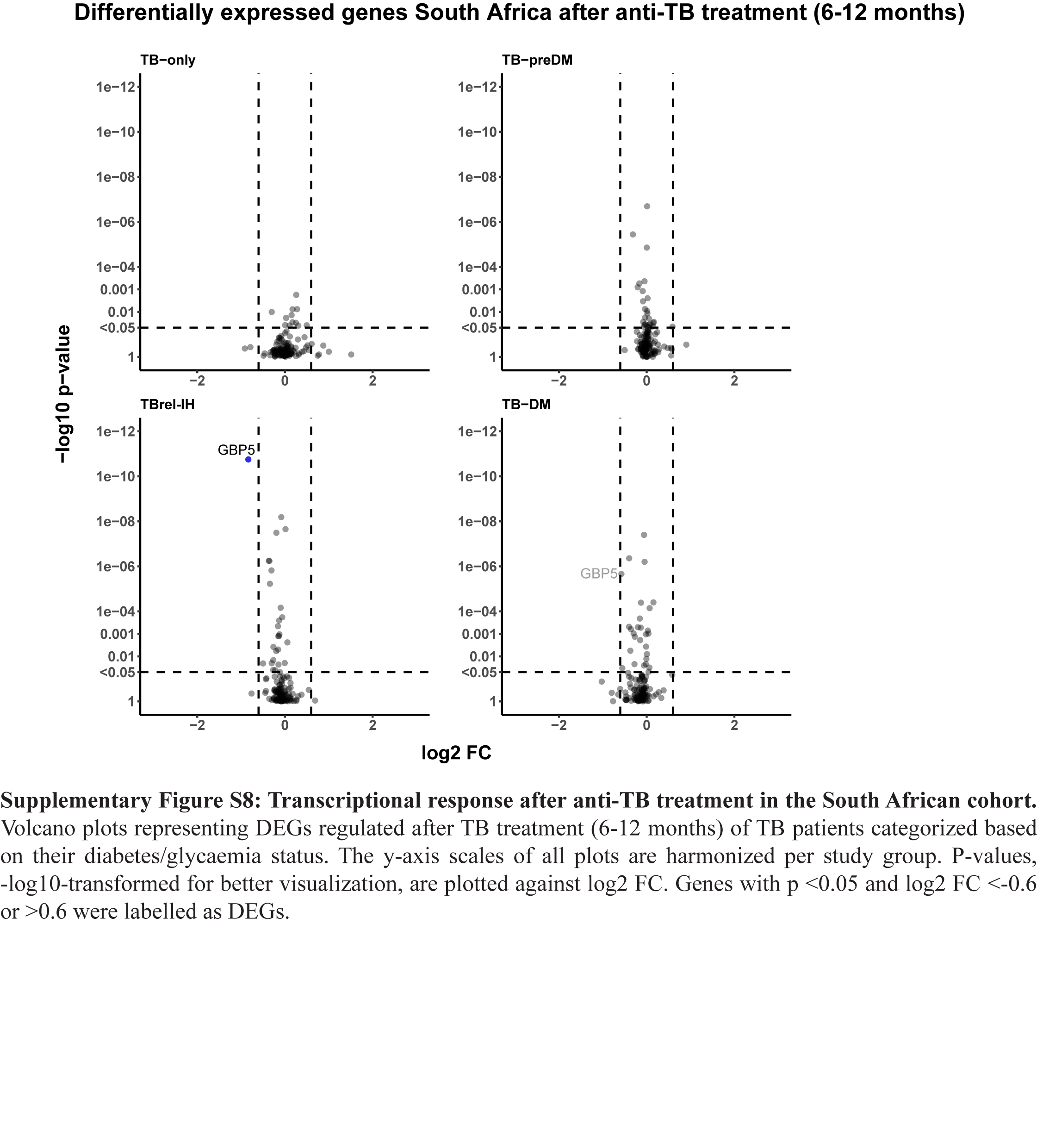

Supplement: Supplementary file 8 — Supporting Information [file CTM2-13-e1375-s009.tif]

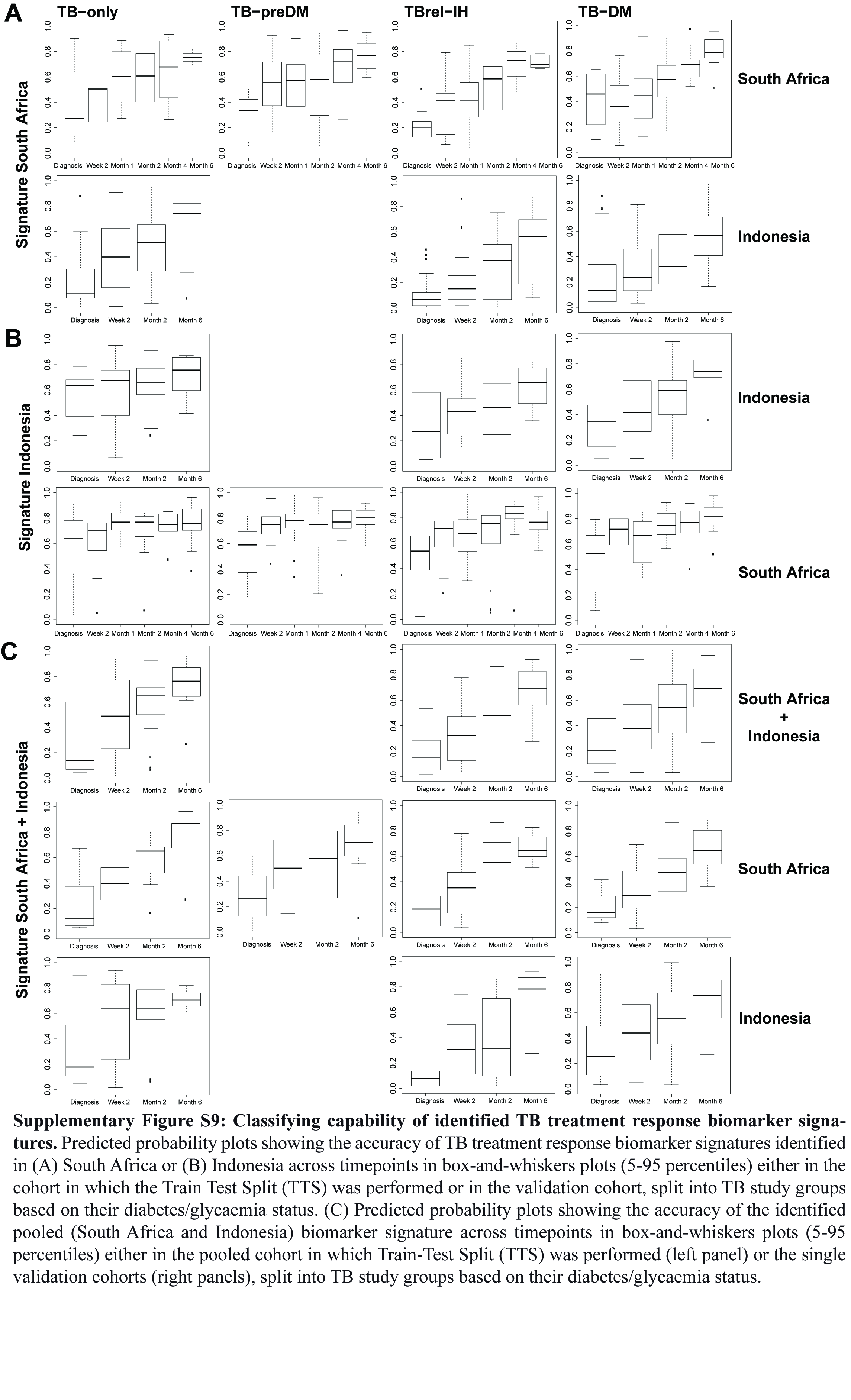

Supplement: Supplementary file 9 — Supporting Information [file CTM2-13-e1375-s018.tif]

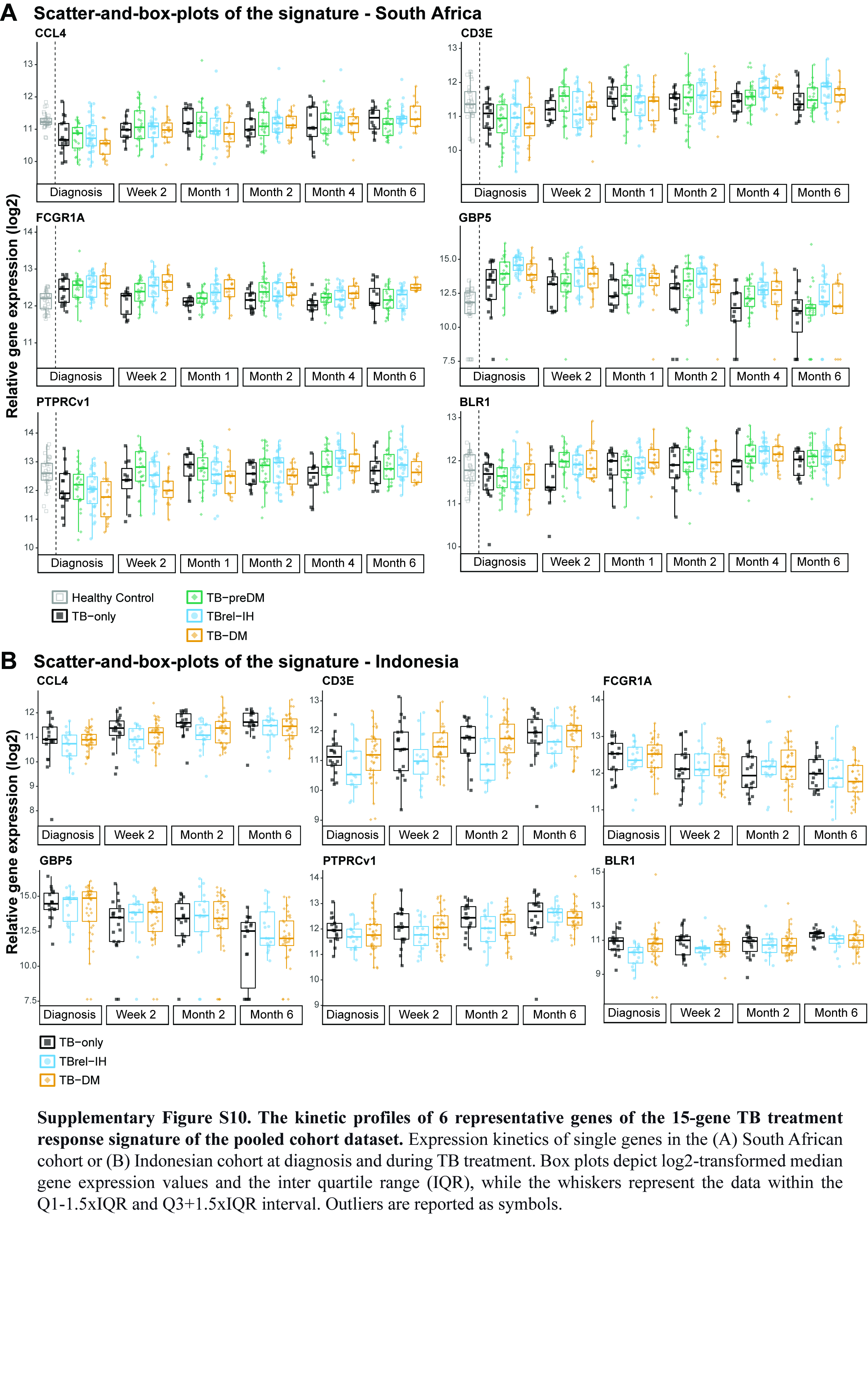

Supplement: Supplementary file 10 — Supporting Information [file CTM2-13-e1375-s010.tif]

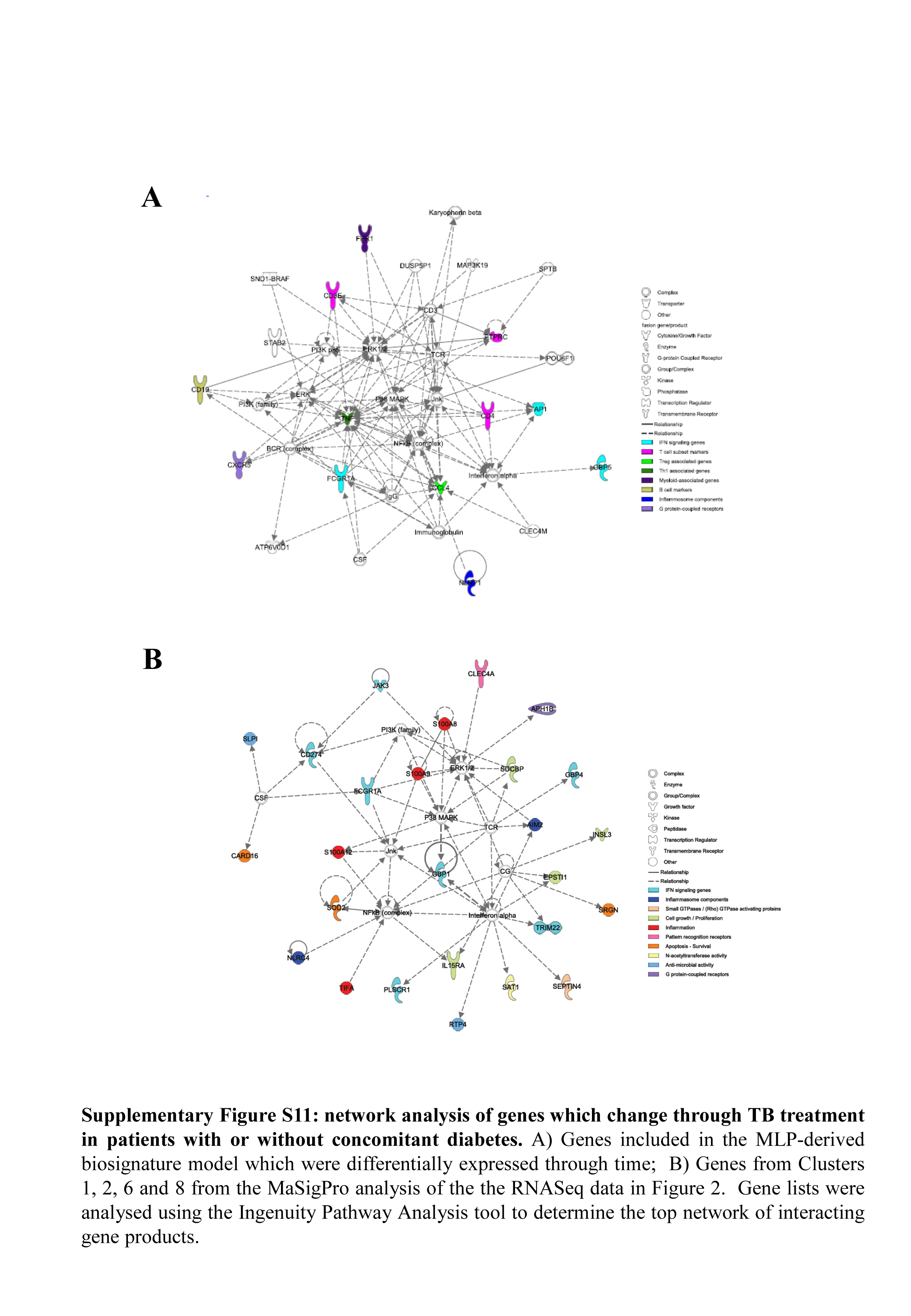

Supplement: Supplementary file 11 — Supporting Information [file CTM2-13-e1375-s021.tif]
